# Expression of CD103 facilitates localization and activation of CD4^+^ T cells within *Mycobacterium tuberculosis* lung-lesions

**DOI:** 10.1016/j.mucimm.2026.02.001

**Published:** 2026-02-03

**Authors:** Thomas Lindenstrøm, Nafsika Panagiotopoulou, Sara B. Cohen, Paula Torres Rodriguez, Joshua S. Woodworth, Mari Morikawa, Mehnaz Halima, Camilla Myhre Maymann, Tu Hu, Sylvia M. Stull, Anders Woetmann, Peter L. Andersen, Kevin B. Urdahl, Benjamin H. Gern, Rasmus Mortensen

**Affiliations:** a Center for Vaccine Research, Department of Infectious Disease Immunology, Statens Serum Institut, Copenhagen, Denmark; b Novo Nordisk Foundation Initiative for Vaccines and Immunity, NIVI Research Center, Department of Immunology and Microbiology, University of Copenhagen, Copenhagen, Denmark; c Seattle Children’s Research Institute, Center for Global Infectious Disease Research, Seattle, WA, USA; d University of Washington, Department of Pediatrics, Seattle, WA, USA; e University of Washington, Department of Global Health, Seattle, WA, USA; f Centre of Vaccinology, University of Geneva, Geneva, Switzerland; g LEO Foundation Skin Immunology Research Center, Department of Immunology and Microbiology, University of Copenhagen, Copenhagen, Denmark; h Department of Infectious Diseases, Novo Nordisk Foundation, Hellerup, Denmark

## Abstract

The spatial localization of CD4^+^ T cells within the *Mycobacterium tuberculosis* (Mtb)-infected lung is critical for optimal immunity. Here, we investigate the role of two E-cadherin binding receptors, CD103 and KLRG1. We demonstrate that KLRG1 restricts CD4^+^ T cells to the lung vasculature early during infection, and limits lesion homing at chronic stages. Subunit vaccination diminishes KLRG1 expression and increases CD103^+^ CD4^+^ T cells associated with improved bacterial control. We identify a link between CD103 expression and Th17 differentiation, as vaccine-induced Th17 cells display increased propensity to upregulate CD103 in the lung. Mixed bone marrow chimeras reveal that CD103 promotes tissue retention and localization of CD4^+^ T cells in close proximity to Mtb, facilitating enhanced TCR signaling. In contrast, CD103-deficient cells remain confined to the lesion periphery with decreased TCR activation. These findings highlight the importance of CD103 in CD4^+^ T cell localization and antigen-sensing with implications for vaccine design.

## Introduction

Tuberculosis (TB), caused by Mycobacterium tuberculosis (Mtb), is a leading cause of morbidity and mortality despite widespread use of the Bacillus Calmette–Guérin (BCG) vaccine.^1^ While BCG provides some protection against severe disease in children, the efficacy in preventing pulmonary TB in adults is limited, and new vaccines with increased lung protection are needed to inhibit transmission and curb the epidemic. Yet, the vaccine pipeline is relatively modest and our incomplete understanding of the mechanisms of protection represents a major impediment for the development and down-selection of improved candidates.

CD4^+^ T cells play a central role in controlling Mtb infection, and depletion of CD4^+^ T cells in both vaccine and non-vaccine settings leads to detrimental outcomes across different animal models.^2–7^ Beyond production of cytokines, the protective capacity of T cells depends on their ability to localize and interact with the infected cells in lung lesions. In that regard, it has been shown that expression of MHC class II is required for optimal bacterial control at the level of individual infected cells, indicating that direct T cell receptor (TCR): MHCII interactions are essential.^8^ However, T cells are often physically segregated from the infected cells in TB lesions, impeding their ability to interact with the infected macrophages.^9–12^ Thus, the ability of CD4^+^ T cells to access and persist in specific microenvironments within infected tissues is crucial for their protective function and a key target for vaccine optimization. Despite this, little is known about the signals guiding CD4^+^ T cell homing, retention and localization within Mtb-infected lung lesions.

Mtb-infection induces distinct T cell populations in the lungs of people with active or previous TB. This includes tissue resident-like memory (T_RM_) T cells characterized by their expression of CD69, CD103, CD49a, and CD44.^13,14^ Through downregulation of the sphingosine-1-phosphate receptor 1 (SIPR1), CD69 prevents cells from re-entering circulation and thereby prolongs peripheral tissue retention.^15^ Among the lung T_RM_ CD4^+^ T cells in people infected with Mtb, the tissue-residency marker CD103 has been reported to be associated with both CD69^+^ and CD69^−^ clusters, whereas expression of the inhibitory receptor KLRG1 appears to be exclusively confined to CD69^−^ clusters.^13^ Both CD103 and KLRG1 are receptors for E-cadherin,^16,17^ which is abundantly expressed by pulmonary epithelial cells within the lung parenchyma.^18^ However, whereas KLRG1 ligation to E-cadherin inhibits effector T cell function through its cytoplasmic ITIM consensus motif,^19–22^ binding of CD103 to E-cadherin promotes adhesion to epithelial cells and/or target cells leading to stabilization of the immunological synapse and improved effector functions.^23–26^ Hence, despite binding to the same receptor, ligation to KLRG1 and CD103 leads to opposing outcomes, which can shape the trajectory of inflammatory responses.^27^ In line with that, CD103^+^ tumor-infiltrating CD8 T cells also correlate with improved prognosis against several different malignancies,^24,28–32^ whereas tumor-infiltration of KLRG1^+^ T cells has been associated with increased tumor burden and progression.^33^

In humans, both CD103^+^ and KLRG1^+^ CD4^+^ T cells have been identified in lung tissues during Mtb infection^13,14^ and overexpression of KLRG1 on peripheral blood CD4^+^ T cells in patients with active TB has been reported.^34^ In mice, KLRG1^+^ CD4^+^ T cells predominantly accumulate in the lung vasculature following Mtb infection,^35–37^ but also constitute an integral part of the Mtb-specific T cell response in the lung parenchyma.^35,36,38,39^ CD103, on the other hand, is almost exclusively found in the lung parenchyma^13,14,36,40^ and has been implicated in the retention of T cells in non-lymphoid tissues, particularly in epithelial barriers.^41^ Hence, CD103-expressing CD4^+^ and CD8 T cell subsets have been reported to be prominent in pleural fluid, bronchoalveolar lavage (BAL) and lung tissue, but not PBMCs, from TB patients,^13,14^ indicating that CD103^+^ T cells may be enriched at the site of infection. In addition, a population of non-vascular CD103^+^ CD4^+^ T cells has been reported after intravenous (i.v.) BCG administration in NHPs.^42^ However, while CD103 has been extensively studied for retaining CD8 T_RM_ cells in solid tumors^29,30,43^ and under homeostatic conditions following cleared viral infections,^44–46^ its significance for the localization and function of CD4^+^ T cells has been understudied and remains largely unexplored for Mtb and other pulmonary infections.^47–49^

In this study, we investigated the role of KLRG1 and CD103 in the localization and activation of CD4^+^ T cells within the Mtb-infected lung. As expected, KLRG1 expressing CD4^+^ T cells are the most frequent population in the infection driven response, both in the lung vasculature and parenchyma, but vaccination reverses this and induces a dominant population of parenchymal CD103^+^ CD4^+^ T cells. Our data suggest that KLRG1 may play an initial role in confining CD4^+^ T cells to the lung vasculature, but also that this is overcome by other mechanisms over the course of infection. In the lung, KLRG1^+^ CD4^+^ T cells are distributed evenly between Mtb lesions and unaffected areas, whereas CD103^+^ CD4^+^ T cells preferentially localize to Mtb lesions, which is further enhanced by vaccination. Importantly, using WT:CD103^−/−^ mixed bone marrow chimeras with multiparameter confocal microscopy, we show that CD103 is required for both the optimal localization and antigen recognition (measured by phosho-S6 (pS6)) of CD4^+^ T cells in proximity to Mtb-infected cells. Together, these data support a previously unrecognized role for CD103 in shaping the spatial distribution and function of CD4^+^ T cells within bacterial microenvironments, which has implications for vaccine design.

## Results

### Mtb infection induces mutually exclusive KLRG1+ and CD103+ CD4^+^ T cell subsets with distinct lung compartmentalization

**Fig. 1.**
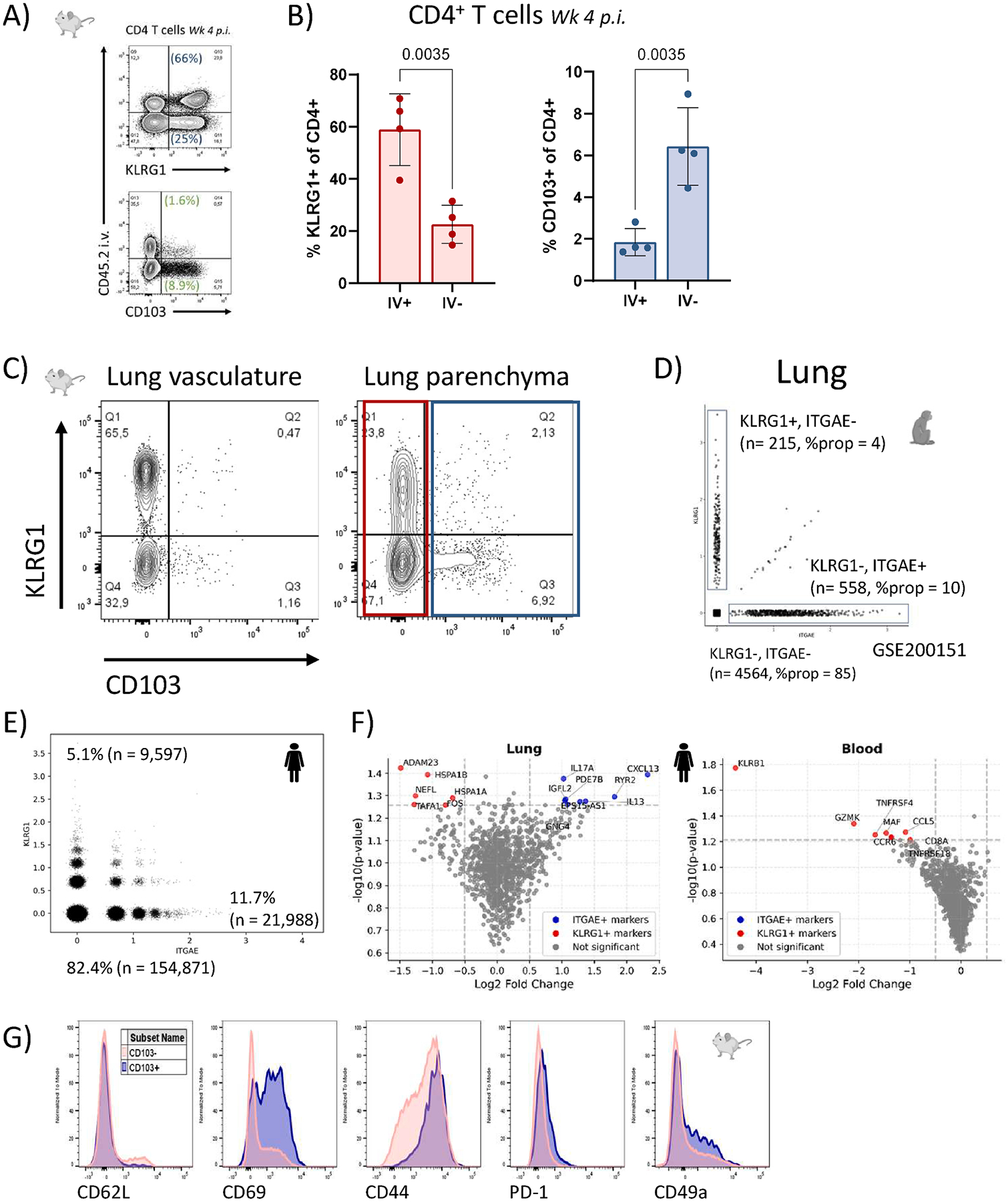
Mtb infection induces mutually exclusive KLRG1^+^ and CD103^+^ CD4^+^ T cell subsets with distinct lung compartmentalization (A-B) Surface expression of KLRG1 (A, upper. B, left) and CD103 (A, lower. B, right) among CD4^+^ T cells in the lung vasculature (IV+ CD45.2) and lung parenchyma (IV− CD45.2) of Mtb-infected C57BL/6 mice at Day 28. p-values calculated using unpaired t-tests. (C) Flow plots depicting surface expression of KLRG1 vs CD103 within lung vasculature (left) and lung parenchymal (right) CD4^+^ T cells at Day 28 post Mtb infection. (D − E) Scatter plot illustrating the relationship between ITGAE (CD103) and KLRG1 expression in CD4^+^ T cells. Each point represents a single cell, with jitter applied to minimize overplotting. Expression levels of ITGAE and KLRG1 are shown as log-transformed counts. Data were obtained from single-cell RNA sequencing of CD4^+^ T cells isolated from MtB infected monkey granulomas at week 10 from the GSE200151 dataset (D) and cells from human resected lung tissues (E), queried from CellxGene. Numbers along axis denote frequencies of resected lung tissue CD4 T cells (F) Volcano plots depicting differential gene expression in lung tissue cells (left) and blood (right) between ITGAE^+^ and KLRG1^+^ T cells. The x-axis shows the log_2_ fold change, and the y-axis represents the −log_10_ (p-value). Genes significantly upregulated in ITGAE^+^ cells (log_2_ fold change > 0.5, FDR-adjusted p < 0.05) are highlighted in blue, and those upregulated in KLRG1^+^ cells are shown in red. Non-significant genes are shown in gray. Dashed horizontal lines indicate the significance threshold corresponding to a false discovery rate (FDR) of 0.05, while vertical dashed lines denote fold change cutoffs. Select top differentially expressed genes are labeled. (G) Surface expression pattern of lung parenchymal CD103^+^ (blue) vs CD103^−^ (red) CD4^+^ T cells at Day 28 post Mtb infection in CB6F1 mice (C57BL/6 × Balb/C). (A-C) Representative data from three similar experiments. (G) One of two similar experiments shown.

We initially set out to study the expression pattern of the two cadherin-binding receptors KLRG1 and CD103 among lung-associated CD4^+^ T cells after murine Mtb infection. In alignment with previous reports,^35–37^ we found that Mtb infection induces a KLRG1^+^ CD4 T cell population with predilection to the lung vasculature (IV+) and a CD103^+^ population that, despite being found in lower frequencies, was primarily tissue-resident and thus confined to the lung parenchyma (IV−) (Fig. 1A, B). Hence, at week 4 post infection, ~60% of the lung vascular CD4^+^ T cells expressed KLRG1 as compared to only ~20% of the parenchymal CD4^+^ T cells. In contrast, CD103 expression among CD4^+^ T cells was significantly more abundant within the lung parenchyma than in the vasculature (6.4% vs 1.8%, respectively). In line with previous findings for tissue resident CD8 T cells,^27,50^ we confirmed that CD103 and KLRG1 define mutually exclusive CD4^+^ T cell subsets during Mtb infection, both within the lung parenchyma as well as in the lung vasculature (Fig. 1C). In order to assess whether their mutually exclusive nature was also a feature of lung granulomas in Mtb-infected primates, we revisited publicly available GSE data from NHP lung granulomas (GSE200151).^51^ Here we found that ITGAE (encoding CD103) was primarily expressed by T cells from isolated granulomas (Suppl Fig. 1A) and its expression was mutually exclusive to KLRG1 in CD4^+^ T cells (Fig. 1D), with no difference among low and high burden granulomas (Suppl Fig. 1B). Similarly, Cellxgene analysis of CD4^+^ T cells from resected human lung tissues across different conditions, revealed expression of KLRG1 and ITGAE largely on distinct cell populations, with CD103-expressing CD4^+^ T cells being more abundant in the lung than KLRG1 expressing CD4^+^ T cells (Fig. 1E). Human ITGAE+ lung tissue CD4^+^ T cells were found to express CXCL13 and IL-17A (Fig. 1F), which have been associated with T cell tissue residency in other settings.^52,53^ Our analysis also showed that ITGAE+ associated markers such as CXCL13 and IL-17A were not significantly expressed within the blood. In line with this, we characterized the parenchymal CD103^+^ CD4^+^ T cell population in mice as CD62L^−^, CD69^+^, CD44^hi^, PD-1^int/+^ and CD49a^int/+^, a staining pattern consistent with tissue resident memory (T_RM_) CD4^+^ T cells (Fig. 1G). Hence, the Mtb infected lung harbors mutually exclusive KLRG1^+^ and CD103^+^ CD4^+^ T cell subsets, with the latter acquiring a T_RM_-like signature.

### KLRG1 and CX3CR1 play compensatory roles in trapping CD4^+^ T cells in the lung vasculature

**Fig. 2.**
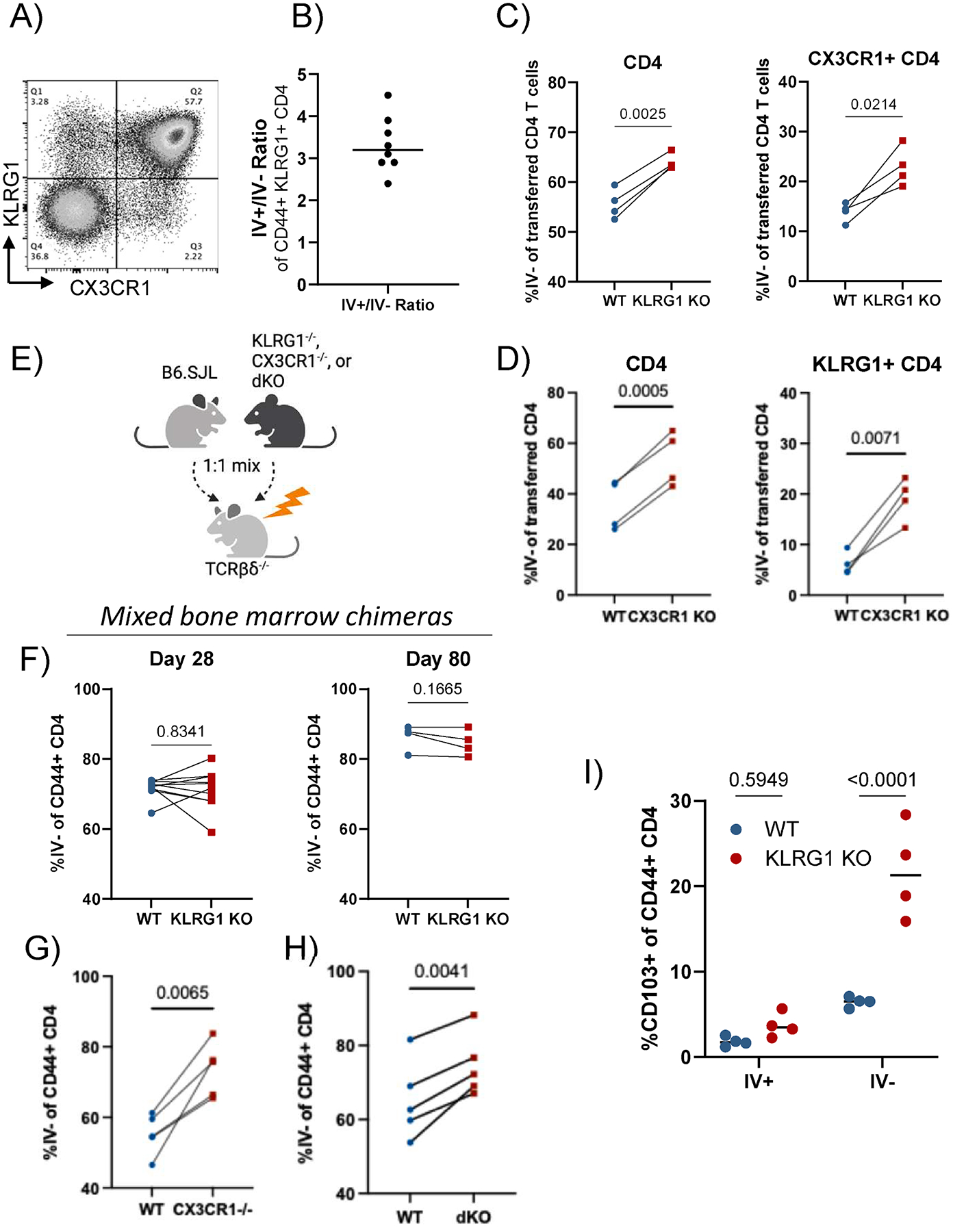
KLRG1 and CX3CR1 play compensatory roles in trapping CD4^+^ T cells in the lung vasculature (A) Surface expression phenotype of lung CD4^+^ T cells isolated from Mtb-infected C57BL/6 mice at Day 28. (B) Ratio of IV+/IV− lung KLRG1^+^ CD44^+^ CD4^+^ T cells isolated from Mtb-infected mice at Day 28. (C-D) Competitive trafficking of adoptively transferred cells. (C) Competitive trafficking of WT vs KLRG1^−/−^ bulk lung CD4^+^ T cells (left) and CX3CR1^+^ lung CD4^+^ T cells (right) to the lung parenchyma 18 h following transfer into Mtb infection-matched (Day 28) recipients. (D) Reciprocal experiment comparing the competitive trafficking of WT vs CX3CR1^−/−^ bulk lung CD4^+^ T cells (left) and KLRG1^+^ lung CD4^+^ T cells (right) following adoptive transfer. (E) Schematic of T cell-specific WT:KO mixed bone marrow chimera generation using sublethal irradiation of TCRβδ^−/−^ mice. (F) Relative localization of WT and KLRG1 KO CD4^+^ T cells to the lung parenchyma (IV−) at Day 28 and Day 80 post-Mtb infection in the mixed chimera setting. (G-H) CD4^+^ T cell localization to the lung parenchyma in (G) WT:CX3CR1 KO mixed chimeras and (H) WT:dKO mixed chimeras at Day 28 post-infection. (I) Expression of CD103 on CD44^+^ lung CD4^+^ T cells from WT and global KLRG1 KO mice at Day 28 post-Mtb infection.

We next investigated the role of KLRG1 in T cell homing. KLRG1 and CD103 both bind to E-cadherin that is abundantly expressed by pulmonary epithelial cells in the parenchyma.^18^ However, KLRG1 also binds to N-cadherin^54,55^ that is expressed on the surface of capillary endothelial cells.^56^ We therefore hypothesized that interactions between KLRG1 and N-cadherin on lung endothelium could ‘trap’ the cells in the capillary bed. This hypothesis is consistent with previous findings that CX3CR1, which is highly co-expressed with KLRG1 on CD4^+^ T cells (Fig. 2A), is also key for inhibiting CD4^+^ T cell entry into the lung.^57^ Indeed, we found that KLRG1^+^ CD44^+^CD4^+^ T cells were approximately three times more numerous in the IV+ relative to the IV− compartment of the Mtb-infected lung (Fig. 1A–B) and (Fig. 2B). To determine the relative contributions of KLRG1 and CX3CR1 in this phenotype, we first sorted CD4^+^ T cells from the lungs of Mtb-infected WT mice and adoptively transferred them into infection-matched, congenically marked WT recipients. Transferred cells were distinguished by the presence or absence of KLRG1/CX3CR1 surface expression, and their ability to reach the IV− lung parenchyma of recipients was assessed 18 h later. Interestingly, KLRG1^+^CX3CR1^+^ CD4^+^ T cells were largely restricted from entering the lung parenchyma, whereas KLRG1^−^CX3CR1^−^ CD4^+^ T cells readily trafficked into the parenchyma, suggesting that these molecules likely tether T cells to the vasculature (Suppl Fig. 2A). We next tested whether genetic deletion of either KLRG1 or CX3CR1 led to a similar phenotype by examining the homing of CD4^+^ T cells isolated from wt vs KLRG1 KO or CX3CR1 KO (identified by knock-in GFP expression) mice. The proportion of transferred cells reaching the parenchyma was found to be significantly higher for KLRG1 KO relative to the co-transferred WT CD4^+^ T cells. The same was seen when gating on CX3CR1^+^ CD4^+^ T cells as a proxy marker for KLRG1^+^ cells, given that they are almost exclusively co-expressed in the IV+ compartment in wt mice (Fig. 2C). This indicates that, even in the presence of CX3CR1, KLRG1 deficiency restricts CD4^+^ T cells from exiting the lung vasculature. Similarly, CX3CR1 KO CD4^+^ T cells (both total and KLRG1^+^) also outcompeted co-transferred WT CD4^+^ T cells, suggesting that both KLRG1 and CX3CR1 contribute to ‘trapping’ T cells in the lung vasculature (Fig. 2D). We next asked whether KLRG1 and CX3CR1 impact T cell homing beyond the tethering phase by establishing WT:KLRG1 KO, WT:CX3CR1 KO, and WT:KLRG1^−/−^ CX3CR1^−/−^ double KO (dKO) mixed bone marrow chimeras (Fig. 2E). Here, in contrast to the dual competitive adoptive transfer experiments (Fig. 2C&D), we found no difference in the capacity of WT and KLRG1 KO CD4^+^ T cells to enter the lung parenchyma at early and late timepoints (Fig. 2F). In contrast, enhanced trafficking of CX3CR1 KO and dKO T cells was observed at day 28 post infection (p.i.) (Fig. 2G&H) as previously reported.^57^ Of interest, and in line with the reciprocal nature of the two markers,^37,58^ we additionally found that CD103 was significantly upregulated on IV− CD44^+^ CD4^+^ T cells in the absence of KLRG1 (Fig. 2I). Taken together, these data suggest that KLRG1 plays an initial role for the confinement of CD4^+^ T cells to the lung vasculature, which is overcome by other mechanisms during the course of Mtb infection, where CX3CR1 appears to play a larger role. In addition, as ablation of KLRG1 was found to significantly increase lung parenchymal CD103^+^ CD4^+^ T cells, KLRG1 expression may impede the transition into CD103^+^ T_RM_.

### Immunization with Ag/CAF^®^ reduces KLRG1 and promotes CD103+ CD4^+^ T cells

**Fig. 3.**
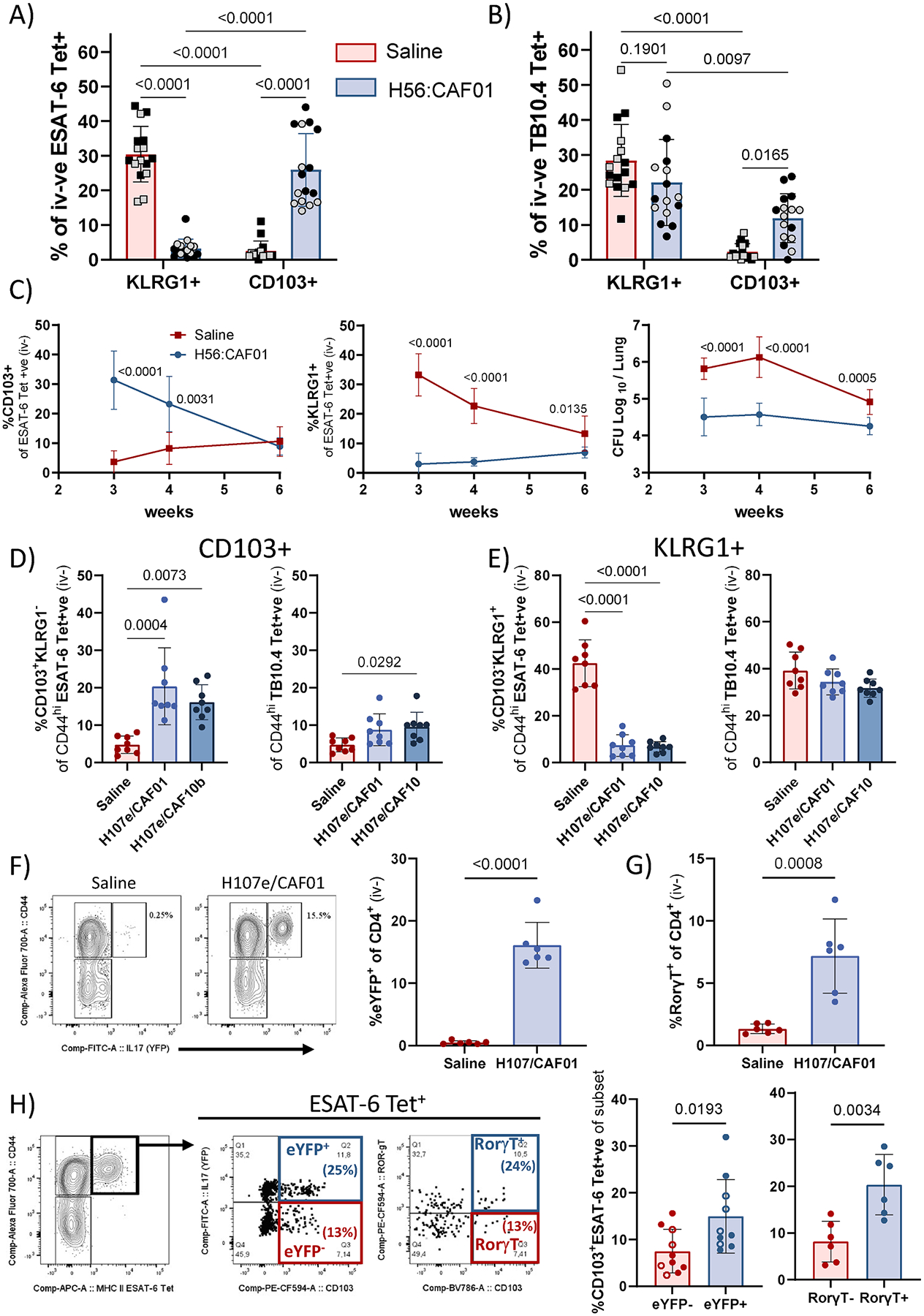
Immunization with Ag/CAF^®^ reduces KLRG1 and promotes CD103^+^ CD4^+^ T cells (A-B) CB6F1 mice (C57BL/6 × Balb/C) were immunized subcutaneously three times with H56/CAF01 or Saline with two weeks intervals and aerosol challenged with Mtb 6 weeks after last immunization. (A) Frequencies of IV-ve, ESAT-6 Tetramer+ ve CD4^+^ T cells expressing either KLRG1 or CD103 at Day 21 post challenge. (B) Frequencies of IV-ve, TB10.4 Tetramer +ve CD4^+^ T cells expressing either KLRG1 or CD103 at day 21 post challenge. Pooled data from two independent experiments (black and grey symbols) with each 8 mice/group (n = 16). Two-way ANOVA with Tukey’s multiple comparison post-test. (C) CB6F1 mice (C57BL/6 × Balb/C) were immunized subcutaneously three times with H56/CAF01 or Saline with two weeks intervals and aerosol challenged with Mtb 6 weeks after last immunization. Frequencies of IV-ve, ESAT-6 Tetramer +ve CD4^+^ T cells expressing either CD103 (left) or KLRG1 (middle) were determined at week 3 (Day 21), 4 (Day 28) and 6 (Day 42) post-challenge. Log10 CFU burdens (right) were determined at the same time points. 6–8 mice per group at each time point. P values determined by multiple unpaired T-tests corrected for multiple comparisons using Holm-Sidak. (D-E) CB6F1 mice (C57BL/6 × Balb/C) were immunized subcutaneously three times with H107e/CAF01, H107e/CAF10b or Saline with two weeks intervals and aerosol challenged with Mtb 6 weeks after last immunization. At Day 28 post-Mtb infection, CD103 (D) and KLRG1 (E) expression was measured among ESAT-6 Tetramer +ve (left) and TB10.4 Tetramer +ve (right) lung parenchymal (IV−) CD4^+^ T cells. Data presented as mean ± SD with data points displaying responses from individual animals (n = 6) (F-H). IL-17A fate reporter mice (IL-17Acre/wtR26ReYFP, in which cells that have transcribed IL17a at any time will continuously express enhanced Yellow Fluorescent Protein (YFP)) were immunized s.c. with H107e/CAF01 two times with four weeks interval and aerosol infected with Mtb 6 weeks later. Lung parenchymal CD4^+^ T cell responses were determined at day 42 post infection. (F) Representative flow (left) and bar plots (right) showing the frequency of lung parenchymal (IV−) Th17 cells (determined by eYFP expression in IL-17A fate reporter mice) in H107e/CAF01 immunized and saline control mice (n = 6). (G) Frequency of lung parenchymal (IV−) Th17 cells as determined by the percentage of CD4^+^ T cells expressing RorgT (n = 6). (H) Flow plots showing lung parenchymal (IV−) ESAT-6 Tetramer+ CD4^+^ T cells in H107e/CAF01 immunized mice at day 42 post Mtb challenge (left) and their CD103 expression relative to eYFP+ vs eYFP− subsets (middle) and RorgT+ vs RorgT− subsets (right). Numbers in parentheses represent the percentage of cells expressing CD103 among the +ve and −ve eYFP and RorgT subpopulations, respectively. Bar plots show the percentages of ESAT-6-specific cells expressing CD103 among the +ve and −ve eYFP subpopulations (left bar plot) and RorgT +ve and −ve subpopulations (right bar plot). Data are presented as mean ± SD with data points displaying responses from individual animals (n = 6; for CD103 expression among eYFP subset, pooled data from two independent experiments are shown (n = 10). P values were in all cases determined by unpaired T-tests (F-H).

Having shown that KLRG1^+^ CD4^+^ T cells dominated the infection-driven response to Mtb, we next investigated how vaccination influenced the expression of CD103 and KLRG1 by comparing H56/CAF01 immunized^59^ and saline control mice. Three weeks post Mtb challenge, we found that H56/CAF01 significantly increased lung parenchymal, vaccine-specific (ESAT-6) CD103^+^ CD4^+^ cells while significantly reducing the frequency of KLRG1^+^ CD4^+^ T cells relative to control mice (Fig. 3A). A significant increase in the proportion of TB10.4 (Esx-H)-specific CD4^+^ T cells (induced by infection and not the vaccine) expressing CD103 was also observed, although this was not as prominent as for ESAT-6 (Fig. 3B). In contrast, KLRG1 expression among the infection-driven TB10.4 response was not significantly different between saline and H56/CAF01-immunized mice (Fig. 3B). The vaccine-effect on KLRG1 and CD103 expression was most striking at week 3p.i., indicating an accelerated CD103 response in the early phases of infection (Fig. 3C, left), whereas KLRG1 expression was less variable and constituted less than 10% of the ESAT-6 tetramer (Tet) specific cells (Fig. 3C, middle). As expected, H56/CAF01 significantly reduced the bacterial burden at all time points (Fig. 3C, right). To investigate if this phenotype translated to other antigens and adjuvants, we next compared CAF01 to the recently developed CAF10b,^60^ using the H107e antigen^61,62^ currently in clinical trials as a TB vaccine candidate (NCT06050356). Consistently, we were able to recapitulate the results observed from H56/CAF01 with H107e/CAF01 and H107e/CAF10b (Fig. 3D–E), concluding that the vaccine-effect on CD103 expressing CD4^+^ T cells in the lung is robust and independent of the vaccine antigen/CAF^®^-adjuvant combination investigated.

### CD103+ CD4^+^ T cells are enriched among vaccine-promoted Th17 cells

We next investigated whether the induction of CD103 was associated with a specific vaccine-induced phenotype. It has been shown that T-bet inhibits the differentiation of CD4^+^ T cells into CD69^+^CD103^+^ tissue-resident cells in the Mtb-infected lung and that its ablation is associated with a dramatic increase in Th17 cells.^37^ Th17 cells have also been described to be T_RM_ precursors during bacterial lung infection^63–65^ and our data-mining of human lung T cells identified IL-17A as one of the differentially expressed genes in CD103+ CD4^+^ T cells (Fig. 1F). We therefore speculated whether the observed increase in CD103 expression after vaccination was linked to Th17-imprinting. To investigate this, we immunized IL-17A fate reporter mice (IL17A^Cre/wt^R26R^eYFP/eYFP^) with H107e/CAF01. In these mice, transcription of IL-17A leads to permanent expression of enhanced yellow fluorescent protein (eYFP), even if IL-17A expression is later lost. As expected, H107e/CAF01 induced robust Th17 lung responses six weeks post Mtb challenge as evident, not only by the significantly induced population of eYFP+ CD4^+^ T cells (Fig. 3F), but also in terms of a significantly higher proportion of parenchymal CD4^+^ T cells expressing RorγT (Fig. 3G). Notably, we found CD103^+^ cells to be significantly overrepresented in the eYFP+ subset relative to the eYFP− subset of the lung parenchymal ESAT-6 Tet+ CD4^+^ T cells, which was also recapitulated in RORγT+ IV-ESAT-6 Tet+ CD4^+^ T cells (Fig. 3H). Together, these findings support that vaccine-promoted Th17 cells have a higher propensity to become CD103^+^ lung resident CD4^+^ T cells during Mtb infection.

### Vaccine-promoted CD103^+^ CD4^+^ T cells are enriched in Mtb infected areas

**Fig. 4.**
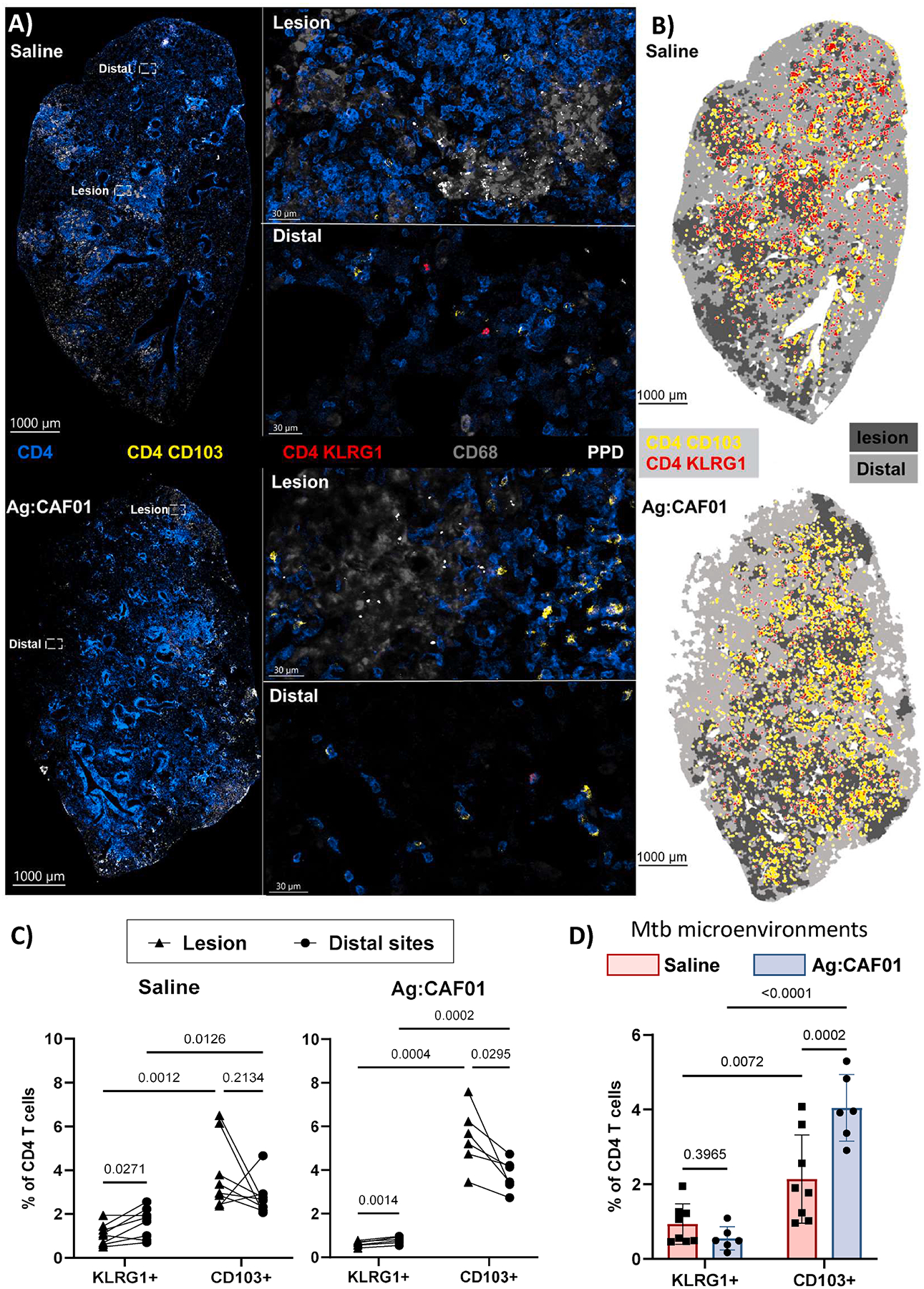
Vaccine-promoted CD103^+^ CD4^+^ T cells are enriched in Mtb infected areas (A-D) C57BL/6 mice were immunized subcutaneously with two doses of Ag:CAF01 or saline, administered three weeks apart, and challenged with Mtb six weeks after the final dose. Lungs were harvested at 21 days p.i (A) Representative confocal microscopy images showing CD4^+^ KLRG1^+^ and CD4^+^ CD103^+^ cells within whole lung sections, with zoom ins of lesions and distal unaffected areas. (B) Positional mapping of CD4^+^ KLRG1^+^ and CD4^+^ CD103^+^ subsets as determined by histo-cytometry. Lesion/distal determined by clustering of microenvironment by cellular composition. (C) Spatial distribution of CD103^+^ and KLRG1^+^ CD4^+^ T cells in lesion areas and unaffected sites in the lung. (D) Frequencies of CD103^+^ or KLRG1^+^ CD4^+^ T cells expressed as a percentage of total CD4^+^ T cells, within Mtb microenvironments, characterized as areas within a 100 μm radius around PPD spots. (C-D) Each sample represents an independent section at least 100um away from other analyzed sections, representing an n of 4 (saline) and 2 (Ag:CAF01) mice. P-values were estimated using Two-way ANOVA.

Studies have identified CD103 expression on CD4^+^ T cells in tumors and other settings of localized inflammation,^47,66^ but its role in homing, retention or positioning of T cells is not well understood. To investigate the spatial distribution of KLRG1 and CD103 expressing CD4^+^ T cells, we performed multiparameter confocal microscopy on lung sections obtained from immunized and saline-treated mice three weeks post Mtb infection. The lung sections were stained with antibodies to identify CD103^+^ and KLRG1^+^ CD4^+^ T cells as well as macrophages (CD68^+^). Additionally, Mtb antigen was identified using an antibody against purified protein derivative (PPD), as previously reported.^12^ Interestingly, CD103+ CD4+ T cells were frequently observed in close proximity to infected macrophages, whereas KLRG1+ CD4+ T cells were not commonly observed in those areas (Fig. 4A). To quantify these observations, we performed histo-cytometry^67^ followed by spatial analysis using CytoMAP.^68^ This approach enabled the phenotypic characterization of distinct cell populations, including CD103^+^ CD4^+^ T cells, KLRG1^+^ CD4^+^ T cells, and Mtb-infected macrophages (PPD^+^CD68^+^) (Suppl Fig. 3), as well as the spatial mapping of these populations within the lung (Fig. 4B). Lesions (dark grey) were defined as regions with increased densities of infected macrophages and CD4^+^ T cells, distinguishing them from unaffected distal sites in the lung (light grey) (Fig. 4B). CD103^+^ CD4^+^ T cells (yellow) and KLRG1^+^ CD4^+^ T cells (red) were mapped to assess their distribution within lesion and distal areas (Fig. 4B) and their densities in each region were quantified (Fig. 4C). Regardless of vaccination status, CD103^+^ CD4^+^ T cells were significantly more abundant within lesion areas relative to KLRG1^+^ CD4^+^ T cells. Additionally, in the immunized group, CD103^+^ CD4^+^ T cells were significantly enriched in lesions compared to unaffected areas (Fig. 4B–C), indicating that immunization promoted accumulation of CD103^+^ CD4^+^ T cells into the lesions. In both groups, KLRG1^+^ CD4^+^ T cells were predominantly localized in unaffected distal sites.

To investigate the interactions between KLRG1^+^ vs CD103^+^ CD4^+^ T cells with infected cells, we next analyzed Mtb microenvironments, defined as regions within a 100 μm radius surrounding PPD spots. As with our lesion-based analysis, CD103^+^ CD4^+^ T cells were significantly more abundant than KLRG1^+^ CD4^+^ T cells within the Mtb microenvironments, regardless of the vaccination status (Fig. 4D). Also, immunization significantly increased the frequency of CD103^+^ CD4^+^ T cells in these areas (Fig. 4A,D). In order to exclude that the increase of CD103^+^ CD4^+^ T cells could just reflect an increase in overall numbers of CD103+ cells in the lung of immunized mice, we normalized the frequency of CD103^+^ CD4^+^ T cells in the Mtb microenvironments to their frequency in the total lung (Suppl Fig. 3C). Even after normalization, we found that vaccination significantly increased the representation of CD103^+^ CD4^+^ T cells within Mtb microenvironments indicating that the effect cannot be explained solely by the global increase of this subset. This suggests that immunization enhances the accumulation of these cells, not only within the lesions, but also in the tight microenvironments surrounding the Mtb bacteria.

### CD103 promotes CD4^+^ T cell localization in lung lesions during Mtb infection

**Fig. 5.**
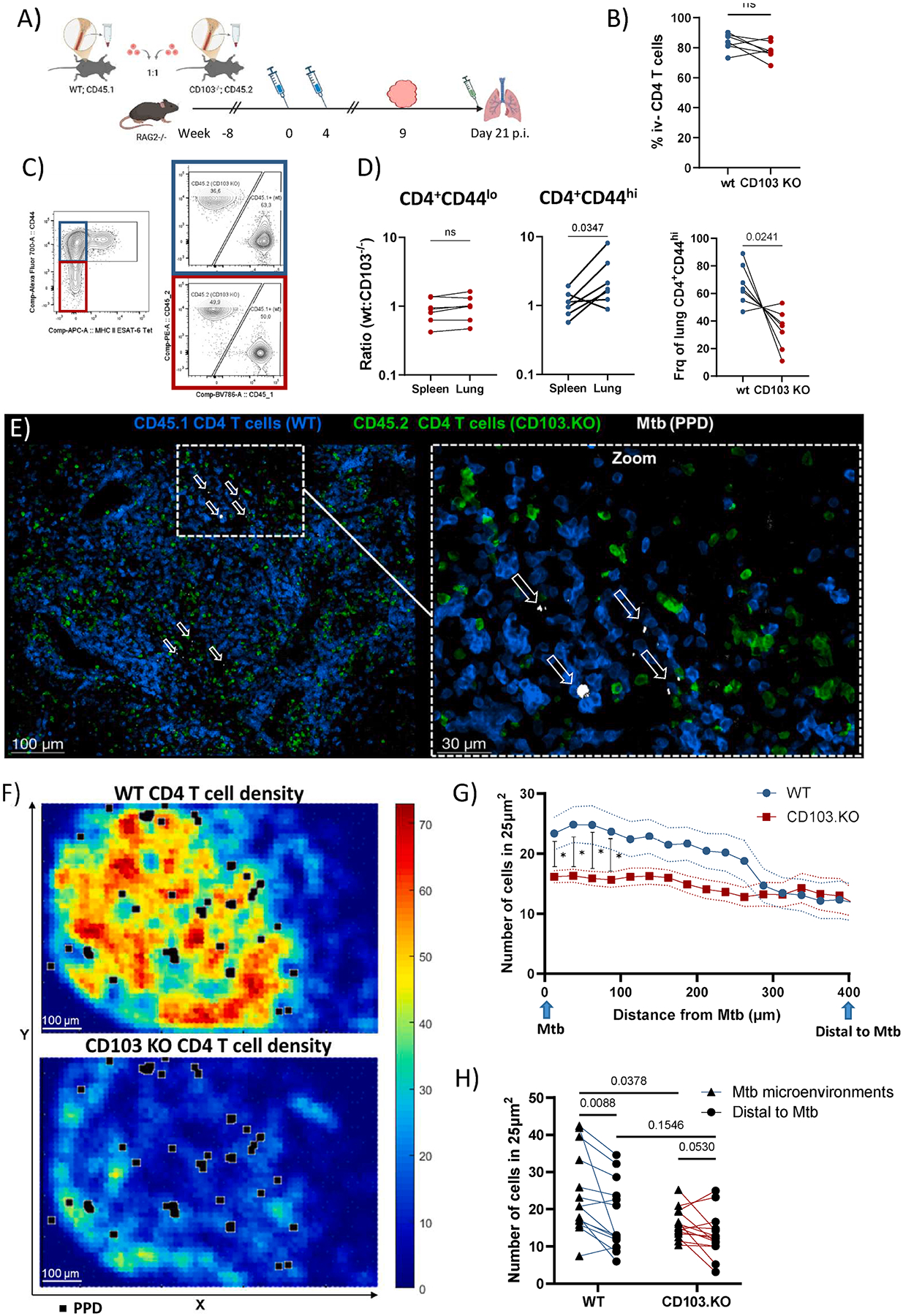
CD103 promotes CD4^+^ T cell localization in lung lesions during Mtb infection (A-G) Mixed BMC mice were immunized subcutaneously eight weeks post reconstitution with two doses of Ag:CAF01 or saline, administered four weeks apart, and challenged with Mtb five weeks after the final dose. Lungs were harvested at 21 days p.i. for flow cytometry analysis and imaging. (A) Schematic representation of the mixed BMC. RAG2^−/−^ mice (CD45.2) were reconstituted in a 1:1 ratio with bone marrow from WT (CD45.1) and CD103^−/−^ (CD45.2) mice and immunized twice using H56/CAF01 with four weeks interval starting eight weeks post reconstitution. Mice were subsequently infected with Mtb by the aerosol route five weeks after last immunization and responses determined at day 21p.i. after a-CD45 i.v. injection. (B) Percentage of WT or CD103^−/−^ lung CD4^+^ T cells that are IV−. Lines connect values within same animals (n = 7) with p-values calculated with a paired *t*-test (C) Gating strategy to determine the percentages of WT (CD45.1) or CD103^−/−^ (CD45.2) cells among lung parenchymal (IV−) CD44^hi^ (blue box) or CD44^lo^ (red box) CD4^+^ T cells. (D) Ratios of WT:CD103^−/−^ cells within the spleen and lung (IV−) were determined among CD44^lo^ (left, red) or CD44^hi^ (middle, blue) CD4^+^ T cell subsets. Lines connect values within same animals (n = 7) and data analyzed with a ratio paired *t*-test. Right graph depicts the frequencies of WT or CD103^−/−^ out of the lung parenchymal (IV−) CD44^hi^ CD4^+^ T cells. Lines connect values within same animals and significance determined by paired *t*-test. (E) Representative confocal image demonstrating WT or CD103^−/−^ CD4^+^ T cells in Mtb areas. White arrows highlight Mtb (PPD staining). For visual clarity, CD4 signal was masked within nuclear objects separately for CD45.1 and CD45.2 signals using Imaris. (F) Representative CD4 density heatmaps of WT (up) or CD103^−/−^ (bottom) CD4^+^ T cells per 25 μm^2^. Black squares represent PPD spots. (G-H) Quantitative microscopy analysis. Each sample represents an image from an individual pulmonary lesion and its surrounding tissue. A total of 14 images were analyzed from 4 mice, with each mouse contributing 3–4 lesions. (G) Quantification of WT or CD103^−/−^ CD4^+^ T cell densities relative to their distance from Mtb bacteria. Cell densities were calculated as the number of cells in 25 μm^2^ across defined radial intervals from any Mtb spot, measured in 25 μm increments. Lines represent mean ± SEM. Statistical significance was determined for each interval using Multiple paired t tests. 0–25 μm (p = 0.044), 25–50 μm (p = 0.033), 50–75 μm (p = 0.038), and 75–100 μm (p = 0.042). For distances beyond 100 μm, p-values were > 0.05. (H) Densities of WT or CD103^−/−^ CD4^+^ T cells in Mtb microenvironments (radius of 0–100 μm from Mtb) or distal to Mtb areas within lesions (radius of 100–400 μm from Mtb). p-values are calculated using Repeated Measures two-way ANOVA with Geisser-Greenhouse correction. Lines connect values within same lesions.

After demonstrating that immunization promoted enrichment of CD103+ CD4^+^ T cells in close proximity to Mtb infected cells, we next asked whether CD103 could be driving T cell retention and localization in these microenvironments. CD103 is expressed by multiple immune cell types, including key subsets of dendritic cells that are central for shaping lung responses,^69^ including against Mtb.^70–72^ Hence, our ability to make firm conclusions about the intrinsic function of CD103 on CD4^+^ T cells during Mtb infection would be compromised in CD103 global KO mice. In addition, we sought to eliminate confounding effects and study CD103-expressing vs -non-expressing CD4^+^ T cell subsets under the same physiological conditions and bacterial burdens. To overcome this, we generated mixed bone marrow chimeras in which lymphocytes, including T cells, were either WT or CD103^−/−^, but virtually all other immune cells derived from the WT host. Rag2 KO mice (CD45.2) were reconstituted with a 1:1 ratio of congenically marked bone marrow from CD103^−/−^ (CD45.2) and WT (CD45.1) mice. Eight weeks after reconstitution, the chimeric mice were immunized twice, four weeks apart and aerosol infected with Mtb 5 weeks later (Fig. 5A). The CD4^+^ reconstitution ratio among PBMCs prior to immunization, after immunization and 3 weeks post Mtb challenge were ~ 1:1 with no significant difference pre- vs post-challenge (Suppl Fig. 4A). Likewise, the ratio in the spleen post-challenge closely reflected the 1:1 reconstitution ratio (Suppl Fig. 4A). In addition, we found no significant difference in the frequency of WT and CD103^−/−^ CD4^+^ T cells that were IV− post Mtb-challenge, suggesting that these genotypes had similar capacity to home to the lung (Fig. 5B). To address whether CD103 played a role in retaining the CD4^+^ T cells that successfully trafficked to lung, we next determined the ratio of lung parenchymal (IV−) WT:CD103^−/−^ T cells. When gating on all CD4^+^ T cells, we observed a trend for increased WT over CD103^−/−^ cells in the lung with an increased ratio of ~ 2.3 (Suppl Fig. 4A). Further subgating on antigen inexperienced cells (Fig. 5C), showed that naïve CD4^+^CD44^lo^ T cells exhibited a ~ 1:1 wt:CD103 KO ratio in both the lung and spleen thus reflecting the reconstitution ratio. This was also the case for antigen experienced CD4^+^CD44^hi^ T cells in the spleen (Fig. 5D left). In contrast, the ratio of WT:CD103 KO cells among the Ag-experienced CD4^+^CD44^hi^ T cells in the lung (iv−) was significantly skewed towards WT cells (with a ratio of ~ 2.8, Fig. 5D middle) with close to 70% of the lung parenchymal CD4^+^CD44^hi^ T cells being of WT origin (Fig. 5D right). This finding supports a role for CD103 in lung retention or accumulation.

To further explore the role of CD103 in CD4^+^ T cell localization within Mtb-infected microenvironments, we analyzed lung tissue from the mixed BMCs using multiparameter confocal microscopy followed by histo-cytometry. First, we confirmed that E-cadherin, the ligand for CD103, was present within Mtb-infected lung tissue in the immunized mixed BMC mice. E-cadherin staining was readily detectable throughout the lung, including within lesion regions and the Mtb microenvironments (Suppl Fig. 5A). Histo-cytometry quantification showed comparable E-cadherin expression within lesions, Mtb microenvironments, and distal lung regions (all p > 0.999), indicating that CD103+ CD4+ T cells have access to their ligand within infected sites (Suppl Fig. 5B). Stochastic events during immunization and infection may preferentially prime or expand certain T cell clones within the chimeric donor pool. Such random effects could alter the relative frequencies of donor-derived cells and potentially confound downstream analyses, especially if Mtb-specific CD4 T cells derive disproportionately from wt or CD103^−/−^ donors. Since MHCII tetramers cannot be used on fixed–frozen tissue, we assessed wt:CD103^−/−^ ratios of tetramer^+^ cells in each chimeric mouse by flow cytometry. These analyses revealed that priming and expansion indeed do occur stochastically, with no consistent directional bias. Hence, in three of four mixed BMCs, individual mice exhibited bidirectional skewing, such that Ag85B- and ESAT-6–specific CD4 T cell responses in a single mouse could be skewed in opposite directions in terms of donor genotypes. (Suppl Fig. 4B–C). As no reproducible, unidirectional skewing was observed among Tet^+^ cells, we next examined how CD103 influences the positioning of CD4 T cells relative to Mtb-infected cells. Confocal imaging showed that WT CD4^+^ T cells frequently formed clusters surrounding PPD stain (Mtb antigen), whereas CD103^−/−^ CD4^+^ T cells appeared more disperse (Fig. 5E). To quantify this spatial distribution, we used CytoMAP to generate density heatmaps, mapping the localization of each population relative to PPD spots (Fig. 5F). Consistent with our microscopy observations, the heatmaps showed that WT CD4^+^ T cells were more concentrated in areas of PPD staining, whereas CD103^−/−^ CD4^+^ T cells were more sparsely distributed in these areas and often confined to the rim of lesions (Fig. 5F). To further quantify these differences, we determined the densities of each cell population relative to their distance from PPD (Mtb antigen) within Mtb lesions (Fig. 5G). Interestingly, we found that the density of WT CD4^+^ T cells was significantly higher within the Mtb microenvironments (<100 μm from any PPD spot) compared to CD103^−/−^ CD4^+^ T cells. This was not the case in lesion areas distal to Mtb, where both populations were found in similar densities (Fig. 5F–H). Notably, the density of WT CD4^+^ T cells increased with closer distances to Mtb infected cells, whereas the density of CD103^−/−^ cells appeared consistent throughout the lesion (Fig. 5F–H). Taken together these data suggest that CD103 expression on CD4^+^ T cells is required for optimal localization of CD4^+^ T cells in the neighborhoods of antigen-bearing cells within Mtb lesions.

### CD103 is required for optimal antigen sensing in Mtb microenvironments

**Fig. 6.**
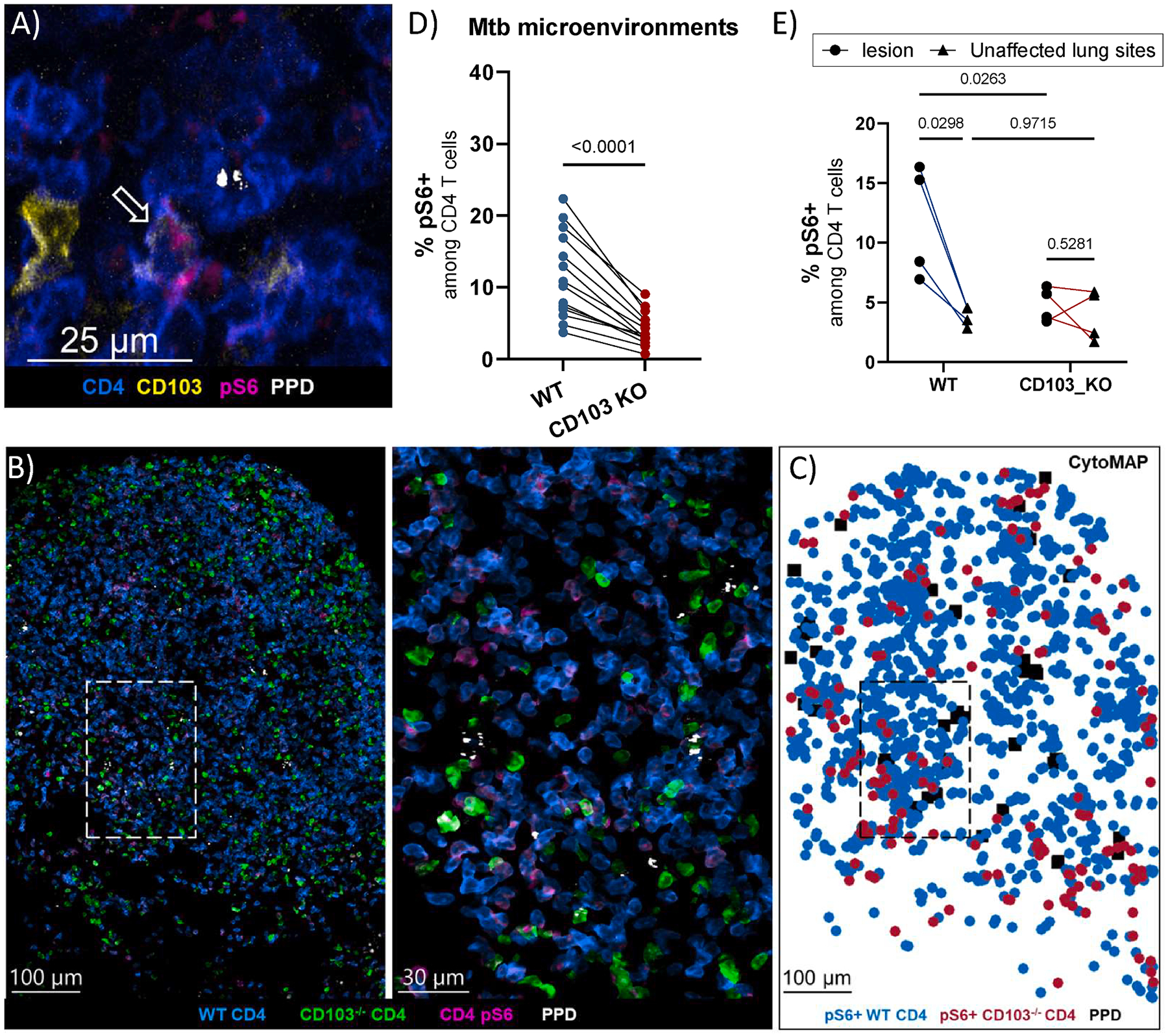
CD103 is required for optimal antigen sensing in Mtb microenvironments (A-E) Mixed BMC mice were immunized subcutaneously with two doses of Ag: CAF01 or saline, administered four weeks apart, and challenged with Mtb five weeks after the final dose. Lungs were harvested at Day 21p.i. for imaging. (A) Representative confocal image showing pS6 staining in the cytoplasm of a CD103^+^ CD4^+^ T cell. (B) Representative confocal image showing pS6+ CD4^+^ T cells among WT or CD103^−/−^ CD4 T cells near PPD spots. (C) Positional mapping (CytoMAP) of pS6+ WT CD4 or pS6+ CD103^−/−^ CD4^+^ T cells in relation to PPD spots. (D) Mtb microenvironments were identified at CytoMap by creating 100 μm-radius Mtb-centered neighborhoods. Graph shows frequencies of pS6+ out of WT or CD103^−/−^ CD4^+^ T cells within Mtb microenvironments. Each data point represents an individual lesion and its surrounding tissue. A total of 14 images were analyzed from 4 mice, with each mouse contributing 3–4 lesions. p-values were calculated with paired t-tests. Lines connect values within same lesions. (E) Frequencies of pS6+ out of WT or CD103^−/−^ CD4^+^ T cells within lesion and unaffected lung sites. Each point represents one mouse (n = 4). For each mouse, lesion and unaffected regions were pooled across images and densities were normalized to region area. p-values are determined using Repeated Measures two-way ANOVA with Geisser-Greenhouse correction. Each data point represents an individual animal. Lines connect values within same animals.

The ability of CD4^+^ T cells to control intracellular Mtb replication is not only dependent on localization, but also requires direct recognition of Mtb infected cells via MHC-II-TCR interactions.^8^ To investigate whether CD103 expression was also associated with increased antigen sensing, we stained lung sections from the mixed BMC mice for ribosomal protein phospho-S6 (pS6), which in T cells is reflective of recent TCR signaling.^39,73,74^ CD103^+^ CD4^+^ T cells expressing pS6 were detected in the Mtb + microenvironments (Fig. 6A), with a significantly higher prevalence of pS6+ cells among the WT CD103^+^ CD4^+^ T cells than WT CD103^−^ CD4^+^ T cells (Suppl. Fig. 6C). To investigate whether CD103 was directly involved in this phenotype, we integrated the staining for pS6 expression with CD45.1 (WT) and CD45.2 (CD103^−/−^) (Fig. 6B). To quantify the observations, we used CytoMAP to identify all pS6+ CD4^+^ T cells within Mtb + microenvironments (<100 μm radius around PPD spots, Fig. 6C). In line with our qualitative observations, this analysis showed that the frequency of pS6+ CD4^+^ T cells was significantly higher among the WT CD4^+^ T cells compared to the CD103^−/−^ cells (Fig. 6D), indicating that CD103 has a vital role in antigen sensing of CD4^+^ T cells within Mtb microenvironments.

It has previously been shown that similar proportions of CD4^+^ T cells display signs of recent TCR activation in Mtb lesions compared to distal regions in the lung, even though Mtb bacteria are only present in the lesions.^39^ This suggests that CD4^+^ T cells are not efficiently sensing antigen at the infection sites during natural Mtb infection, which may constitute an important barrier for optimal CD4^+^ T cell mediated protection. Having shown that vaccination increases CD103 expressing CD4^+^ T cells (Fig. 3) and that CD103 promotes optimal T cell localization and activation, we finally asked whether pS6 would be differentially expressed in affected vs. unaffected tissues in the context of vaccination. Indeed, histo-cytometry analysis of lesions and unaffected lung sites revealed that the frequency of pS6+ WT CD4^+^ T cells was significantly elevated within Mtb-lesions, an effect that was most pronounced in the CD103^+^ WT cells (Fig. 6E, Suppl Fig. 6C) whereas pS6 was comparable between lesion and unaffected areas for CD103^−/−^ cells. Together this indicates that vaccination may enhance antigen sensing in Mtb-lesions through a CD103-dependent mechanism.

## Discussion

Direct recognition of Mtb-infected macrophages by CD4^+^ T cells is a requirement for optimal intrinsic control of Mtb.^8^ Consequently, understanding how T cells migrate to, are retained within, and strategically localize inside Mtb lung lesions is crucial.

In the present study we focus on the significance of CD4^+^ T cells expressing KLRG1 and CD103 – two receptors that both recognize E-cadherin, but where ligation leads to disparate outcomes. Whereas KLRG1 is associated with terminal differentiation in Mtb and can lead to exhaustion,^21,34,37,75–77^ CD103 is linked to tissue residency^13,14,78,79^ with signaling leading to improved effector functions, notably among tumor-infiltrating CD8 T cells,^24,28–30^ but also among CD4^+^ T cells.^47,48,80^ In line with previous mouse studies, we initially show that KLRG1^+^ CD4^+^ T cells are a dominant population during Mtb infection,^35–38,81^ and that CD103 and KLRG1 expression delineates two distinct and mutually exclusive CD4^+^ T cell populations. When revisiting publicly available data from lung tissues, we find that their mutually exclusive nature is not only a trait of TB lesions in mice, but is also seen in NHP lesions and human lung tissue CD4^+^ T cells, which suggests that this is a conserved attribute.^27,50^ In line with other studies,^13,47,49,82^ we found that CD103^+^ CD4^+^ T cells express several markers associated with T_RMs_, indicating that they may have a preference to accumulate in peripheral tissues. In contrast, activated KLRG1^+^ CD4^+^ T cells were observed to occur three times more often in the lung vasculature than in the parenchyma.

In short-term dual competitive adoptive transfer experiments, we reveal a role for KLRG1, along with CX3CR1, in the confinement of CD4^+^ T cells to the lung vasculature. Yet, although vascular tethering could be a plausible explanation for the observed effect in these short-term adoptive transfer models, other factors that may alter the ability of the cells to enter the lung could still be involved. Our mixed BMC data also suggests other mechanisms to be at play, as the role of KLRG1 seem to be overridden by other mechanisms in long-term studies. These mechanisms may include increased luminal expression of CX3CL1 (the receptor for CX3CR1) on inflamed endothelia, which could favor vascular retention through CX3CR1-dependent mechanisms during chronic Mtb settings. Based on this, and the study by Hoft *et al*.^57^ that used anti-KLRG1 blocking antibodies, we conclude that KLRG1 is negligible for inhibiting long-term transendothelial diapedesis into the lung parenchyma. However, KLRG1-deficiency led to increased parenchymal expression of CD103, suggesting that arresting highly differentiated Th1 states could support transition into CD103^+^ T_RM_, as also shown after downregulation or full ablation of T-bet.^37,58^ Although not specifically tested in CX3CR1 KOs, we expect that ablation of such markers that limit CD4 T cell egress from the vasculature would cause greater CD4 T cell numbers to reach the lung parenchyma, eventually leading to a similar proportion expressing CD103.

Vaccination represents an attractive means to manipulate T cell responses, and in this paper we observed that vaccination with either H56/CAF01^59^ or H107e/CAF10b,^60,61^ which is currently in clinical trials (NCT06050356), significantly increased CD103 expressing CD4^+^ T cells in the lung parenchyma, while diminishing KLRG1^+^ CD4^+^ T cells. These changes were associated with reduced CFU levels. As this trait was independent of the antigen used (H56 vs H107e) and most strikingly observed among vaccine-promoted (ESAT-6) responses compared to infection-driven responses (TB10.4), we conclude that this is unlikely to be attributed to the infectious load, and rather may be imprinted by the vaccine adjuvant. In that regard, we asked whether there was a link between CD103 expression and induction of vaccine-specific Th17 cells, which is a hallmark of the parenterally administered CAF^®^ adjuvants used in this study.^83,84^ Although CD103 expressing ESAT-6 Tet+ CD4^+^ T cells were not exclusively confined to Th17 subsets, we found that CD103^+^ cells were significantly overrepresented in vaccine-imprinted Th17 lineages regardless of whether they were identified by Th17 fate-mapping eYFP-expression or by the transcription factor RORγT. Of interest in this context, we observed that human ITGAE+ lung CD4^+^ T cells express IL-17 (Fig. 1F), which is in line with studies showing that CD103+ T cells express cytokines associated with Th17/Tc17 cells in other inflammatory contexts.^27,64,65^ Together, these observations suggest that Th17 cells may be precursor cells with a preferential capacity to express CD103 and/or that Th17 cells have a propensity to localize to the lesions, potentially through unique combinations of chemokine receptors and integrins, where they upregulate CD103 in response to tissue-specific stimuli. These mechanisms are not mutually exclusive, but we speculate that tissue-specific cues, such as lesion-localized TGFβ,^39^ play a critical role for the induction of CD103 expression, as has been described in other settings.^27,63,85,86^

Although CD103 is associated with T_RMs_, both CD103^+^ and KLRG1^+^ T cells seed the Mtb infected lung. However, whether these two subsets differ in their spatial organization was previously unknown. Here, we report that CD103^+^ CD4^+^ T cells were significantly more abundant within lesions and in particular in the critical microenvironments surrounding the antigen-bearing cells. In contrast, KLRG1-expressing subsets were largely relegated to unaffected distal sites. While this was seen regardless of vaccination status, Ag/CAF immunization significantly enhanced the accumulation of CD103^+^ CD4^+^ T cells in these areas, also after normalization to account for the global increase of this subset in the lung among immunized mice. Hence, the accumulation of CD103^+^ CD4^+^ T cells in the Mtb microenvironments most likely reflects a combined effect of vaccination expanding their precursor subset and CD103^+^ cells being predisposed to localize in the infected sites. This may suggest that CD103 has a direct role in T cell homing, but mixed BMC showed no difference in the capacity of WT and CD103^−/−^ CD4^+^ T cells to enter the lung parenchyma. This is in line with observations from other tissue sites, showing that CD103 is dispensable for the entry into gut and skin tissue^85,87^ and therefore was interpreted to mean that CD103 does not serve as a homing receptor. Instead, we discovered that CD103 plays a significant role in retaining CD44^+^CD4^+^ T cells in the lung parenchyma, as WT cells were close to 3 times more abundant than their CD103^−/−^ counterparts, which is in line with reports from skin resident CD103^+^CD8^+^ T_RMs_.^85^

As a major finding of this study, we also found that the density of WT CD4^+^ T cells within 100um of Mtb antigen was significantly higher compared to CD103^−/−^ CD4^+^ T cells. This was not the case in lesion areas distal to Mtb, where both populations were found in similar densities. In addition, the density of WT cells was found to increase with closer distances to Mtb, whereas the density of CD103^−/−^ cells remained consistent throughout the lesion. This demonstrates that CD103 is implicated in retention and localization of CD4^+^ T cells in microenvironments surrounding areas of high infection burden, which render it more likely for an Mtb-specific CD4 T cell to encounter Ag within that lesion. This provides novel insights on the role of CD103 in CD4^+^ T cell lung localization with our findings being in line with observations from several cancers, where CD103 expression has been identified as a key feature for tumor infiltrating lymphocytes.^29,31,47^ Likewise, in psoriasis patients, CD103^+^ T_RMs_ are highly enriched in active and resolved psoriasis lesions compared to non-lesional skin sites from the same patients.^65^

As a potential mechanism for the observed accumulation of CD103^+^ CD4^+^ T cells, we speculate that CD103^+^ CD4^+^ T cells increase their avidity for E-cadherin once they receive cognate TCR engagement and tissue-specific stimuli via TGF-β, which is highly expressed in areas close to Mtb.^39^ This is based on studies with CD8 T cells, showing that such combined TCR/TGFβR1 stimulation promotes ‘inside-out’ signaling, resulting in a conformational change of the extracellular domain of αeβ7 leading to increased avidity of binding between CD103 and E-cadherin.^17,28,88^ Accordingly, intratumoral positioning of CD8 tumor infiltrating lymphocytes (TILs) has been shown to directly involve CD103 through initiation of such ‘inside-out’ signaling.^28^ If this happens in the Mtb lung environments, where we find E-cadherin available for CD103-mediated ligation (Suppl Fig. 5), the triggered increase in avidity for E-cadherin could “lock” the CD4^+^ T cells at the right location and enhance their antigen sensing. In support of this, we found that pS6 expression, as a marker for TCR signaling, was significantly more frequent among the WT compared to the CD103^−/−^ CD4^+^ T cells. Furthermore, the frequency of pS6+ WT CD4^+^ T cells was significantly elevated within Mtb-lesions compared to unaffected lung tissue, which was not the case for CD103^−/−^ cells, where pS6 expression did not differ between lesions and unaffected sites. This shows that CD103 increases antigen sensing, which could either occur through positioning, “locking” of the CD4^+^ T cells near the antigen presenting cells and/or by lowering the threshold for TCR stimulation. In this matter, ‘outside-in’ signaling through CD103 has been shown to act either as a positive co-stimulatory molecule^48,78^ or to directly accentuate signaling through the TCR.^26^

Together, we uncover a role for CD103 expression in the retention, localization, and enhanced antigen sensing of CD4+ T cells in proximity to Mtb antigen within lung lesions. This identifies a potential mechanism for overcoming a key barrier to effective CD4^+^ T cell–mediated immunity against TB. We further demonstrate that vaccination with a candidate currently in clinical trials can selectively induce lung parenchymal CD103+ CD4 T cells, and we propose that CD103-dependent localization and enhanced activation, together with the continual recruitment of KLRG1– CD4 T cells,^35,89^ contribute meaningfully to the protection afforded by the Ag:CAF01 vaccine. Thus, our findings highlight CD103+ CD4 T cells as an important focus for future studies of vaccine-induced protection and inform the rational design of next-generation TB vaccines.

## Materials and methods

### Mice

The following mouse strains were used: Statens Serum Institut (SSI): C57BL/6JOlaHsd and CB6F1 (C57BL/6 × Balb/c) mice were obtained from ENVIGO; C57BL/6NCrl and B6.SJL-Ptprc^a^Pepc^b^/BoyCrl (CD45.1 wt; # 494) from Charles River, RAG2 KO (B6.129S6-*Rag2*^*tm1Fwa*^ N12) from Taconic. Cryo-recovered breeding pairs of CD103 KO mice (B6.129S2(C)-*Itgae*^*tm*1*Cmp*^/J; Strain #006144; CD45.2) were purchased from The Jackson Laboratory (Bar Harbor, USA) and subsequently bred for homozygous deletion of CD103 at SSI. IL-17A fate reporter mice (IL-17a^Cre/wt^ Rosa26R^eYFP/wt^) were bred and handled at the experimental animal facility at SSI and produced by crossing the Il17a^tm1.1(icre)Stck^/J (IL-17cre) strain with the B6.129X1-Gt(ROSA) 26Sor^tm1(EYFP)Cos^/J (R26R-EYFP) strain, both purchased from The Jackson Laboratory. Seattle Children’s Research Institute (SCRI): C57BL/6, CX3CR1-GFP: Jackson Labs # 5582. These were also crossed to B6.SJL-Ptprc^a^Pepc^b^/BoyJ to create a CD45.1 expressing KO and further crossed to KLRG1^−/−^ to create a dKO line. The KLRG1 KO mice were a kind gift from Joanne Turner (The Ohio State University). TCRbd mice were purchased from Jackson Labs and maintained as a colony in the SCRI facility.

At SSI, mice were housed at specific pathogen-free conditions and randomly assigned to cages of eight. Before the initiation of experiments, mice had at least 1 week of acclimatization in the animal facility. During the course of the experiment, mice had access to irradiated Teklad Global 16% Protein Rodent Diet (Envigo, 2916C) and water ad libitum. Mice were housed at an ambient temperature of 20–23 °C and 45–65% relative humidity on a 12 h/12 h light/dark cycle with 15 min dusk and dawn transition periods under Biosafety Level (BSL) II or III conditions in individually Type III ventilated cages (Scanbur, Denmark) and had access to nesting material (enviro-dri and soft paper wool; Brogaarden) as well as enrichment (aspen bricks, paper house, corn, seeds, and nuts; Brogaarden). Animals were monitored and handled by full-time staff and non-aversive handling (such as cup- and tunnel-handling) used whenever possible. At SCRI, mice were housed in specific pathogen-free conditions in individually ventilated cages (maximum density of 5 mice per cage) with negative pressure ventilation and air filtering). Animals were monitored under care of full-time staff, given access to food and water ad libitum and maintained under a 12-hour light/dark cycle, with temperature maintained at 22–25 degrees Celsius. All were of normal health and immune status, and were treatment, procedure, and invasive testing naïve prior to the initiation of our studies.

#### Ethics for animal studies.

Statens Serum Institut’s Animal Care and Use Committee (IACUC) approved all experimental procedures and protocols carried out at SSI. All experiments were conducted in accordance with the regulations put forward by the Danish Ministry of Justice and Animal Protection Committees under license permit no. 2019-15-0201-00309 and in compliance with the European Union Directive 2010/63 EU. At SCRI, experiments were performed in compliance with the SCRI Animal Care and Use Committee.

#### Fusion proteins & Immunization regimens.

H56 and H107e fusion proteins used in the current study were produced and purified as previously described.^62^ H56 is a fusion protein consisting of Ag85B-ESAT-6-Rv2660c, whereas H107e is a fusion of the eight proteins PPE68, ESAT-6 (four intercalating copies), EspI, EspC, EspA, MPT64, MPT70 and MPT83 Mice were immunized subcutaneously (s.c.) at the base of the tail, with a volume of 200 μl. Mice were either immunized three times with two-week intervals (week 0, 2, and 4) or twice with a four-week interval as indicated in the figure legends. H56 (5 μg per dose) and H107e (2 μg per dose) were diluted in Tris-HCL buffer + 2% Glycerol (pH 7.2) and formulated in either Cationic Adjuvant Formulation 1^®^ (CAF^®^01) composed of 250 μg DDA / 50 μg TDB per dose or Cationic Adjuvant Formulation 10b^®^ (CAF^®^10b) composed of 250 μg DDA / 50 μg MMG / 20 μg ODN2006 per dose. Negative control mice were immunized with Tris-HCL buffer only or left non-vaccinated.

#### Mycobacterial infections.

At SSI, mice were aerosol challenged with M.tb Erdman strain (ATCC 35801/TMC107) 5–6 weeks after last immunization, which represents an early memory response after the main contraction of effector cells peaking at week 2–3 post immunization.^38,83,90^ Mtb Erdman was cultured in Difco^™^ Middlebrook 7H9 (BD) supplemented with 10% BBL^™^ Middlebrook ADC Enrichment (BD) for two–three weeks using an orbital shaker (~110 rpm, 37 °C). Bacteria were harvested in log phase and stored at −80 °C until use. On the day of the experiment, the bacterial stock was thawed, sonicated for five minutes, thoroughly suspended with a 27 G needle, and mixed with PBS to the desired inoculum dose. Mice were aerosol-challenged with virulent Mtb Erdman in a dose equivalent to 50–100 CFUs using a Biaera exposure system controlled by AeroMP software. At SCRI, all Mtb infections were done as previously described.^39^ Briefly, a stock of Mtb H37Rv was aerosolized using a Glas-Col aerosol infection chamber adjusted to deposit 50–100 CFU directly into the lungs of mice enclosed.

#### Enumeration of Mtb in organs.

In order to determine vaccine efficacy, Mtb CFU were counted in lungs and spleens. Using GentleMACS M-tubes (Miltenyi Biotec), left lung lobes or spleens were homogenized in 3 mL MilliQ water containing PANTA^™^ Antibiotic Mixture (BD, cat. no. #245114). Tissue homogenates were serially diluted before being plated onto 7H11 plates (BD) and grown for 14–21 days at 37 °C and 5% CO2. CFU data were log-transformed before analyses.

#### Preparation of single-cell suspensions.

Spleens, lungs, inguinal lymph nodes were aseptically harvested from euthanized mice and processed to extract single cell suspensions as previously described.^62^ Briefly, lungs were first homogenized in Gentle MACS tubes C (Miltenyi Biotec), followed by 45 min collagenase digestion (Sigma Aldrich; C5138) at 37 °C, 5% CO_2_. The lung homogenate, spleens, and lymph nodes were subsequently forced through 70-μm cell strainers (BD) with the plunger from a 3 mL syringe (BD). Cells were washed twice in cold RPMI or PBS followed by 5 min centrifugation at 700 × *g*. Finally, cells were resuspended in supplemented RPMI media containing 10% fetal calf serum (FCS). PBMCs were purified by centrifugation on Lympholyte (Cederlane). Cells were counted using an automatic Nucleocounter (Chemotec) and cell suspensions were subsequently adjusted to 1–2 × 10^6^ cells/well for flow cytometry.

### Flow cytometry

#### Tetramer staining for flow cytometry analyses

Class II MHC Tetramers (I-A^d^:TB10.4_73–88_, I-A^b^:ESAT-6_4–17_) conjugated to BV421, PE or APC and corresponding negative controls (I-A^d^: hCLIP, I-A^b^:hCLIP) were provided by the NIH tetramer core facility (Atlanta, USA). Single-cell suspensions were stained with tetramers diluted 1:100 in FACS buffer (PBS + 1%FCS) containing 1:100 Fc-block (anti-CD16/CD32) and fixable viability dye eFlour^™^506 (1:500) or eFlour^™^780 (1:500) (both eBioscience) for 30 min at 37 °C, 5% CO_2_. Tetramer staining was followed by surface staining, fixation, and eventually transcription factor staining as described below.

#### Surface and intracellular staining

Cells were stained for surface markers diluted in 50% brilliant stain buffer (BD, 566349) at 4 °C for 30 min. Cells were then fixed and permeabilized using the Foxp3/Transcription Factor Staining Buffer Set (eBioscience^™^; 00-5523-00) as per manufacturer’s instructions followed by RORγT staining for 30 min or overnight at 4 °C. Fluorescence minus one controls were performed to set gate boundaries for selected markers. Cells were acquired on a BD LSRFortessa and the FSC files were manually gated with FlowJo v10 (Tree Star). The following antibodies were used: CD3-BV605 (BioLegend, clone: 17A2, catalog #100229), CD4-BV510 (Biolegend, clone: RM4.5, catalog #100559), CD4-BV786 (BD biosciences, clone: GK1.5, catalog #563331), CD8-PerCP-Cy5.5 (eBiosciences, clone: 53–6.7, catalog #45–0081–82), CD8 BV650 (BD biosciences, clone 53–6.7, catalog #563234), CD44 AF700 (Biolegend, clone IM7, catalog # 103026), CD44 BV786 (BD biosciences, clone IM7, catalog #563736), CD45-FITC (Biolegend, clone: 30-F11, catalog #103108), CD45.1 BV785 (Biolegend, clone A20, catalog #110743), CD45.2-FITC (BD biosciences, clone: 104, catalog #553772), CD45.2-PE (BioLegend, clone: 104, catalog #109808), CD45.2-PE-Cy7 (BD biosciences, clone 104, catalog #560696), CD49a-PE-Cy7 (BioLegend, clone: HMα1, catalog #142608), CD49a-BV421 (BD bioscience, clone HMα1, catalog # 740046), CD49a-BV711 (BD biosciences, clone: Ha31/8, catalog #564863), CD62L-APC-R700 (BD biosciences, clone: MEL-14, catalog #565159), CD69 PE (BD bioscience, clone: H1.2F3, catalog #561932), CD69 PE-Cy7 (BD bioscience, clone: H1.2F3, catalog #552879), CD103-PEDazzle594 (BioLegend, clone: 2E7, catalog #121430), CD103-PE-CF594 (BD bioscience, clone: M290, catalog #565849), CD103 BV785 (BioLegend, clone: 2E7, catalog #121439), KLRG1-FITC (eBioscience, clone: 2F1, catalog #11-5893-82), KLRG1-PE-Cy7 (eBioscience, clone: 2F1, catalog #25-5893-82), KLRG1-APC-Cy7 (BioLegend, clone: 2F1, catalog #138426), KLRG1-BV711 (BioLegend, clone: 2F1, catalog #138427), KLRG1-BV711 (BD biosciences, clone: 2F1, catalog #564014), Live/dead-eF780 (eBioscience, catalog #65-0865-18), Live/dead-e506 (eBioscience, catalog #65-0866-18), PD1 BV605 (Biolegend, clone: 29F.1A12, catalog #135220), RorgT PECF594 (BD bioscience, clone: Q31–378, catalog #562684), CD3 BV605 (BD bioscience, clone: 145–2C11, catalog #563004, Biolegend, clone 17A2, catalog #100237), CD3 BV650 (Biolegend, clone 17A2, catalog #100229), CD3 PerCp (BD bioscience, clone 145–2C11, catalog #561089), CD19 BV510 (Biolegend, clone: 6D5, catalog #115545), CD19 PerCp (BD bioscience, clone: 1D3, catalog # 551001).

### Immunofluorescence staining and confocal microscopy

Mouse lungs were harvested and immersed in BD Cytofix diluted with PBS in a 1:3 ratio, for 24 h at 4 °C. The lungs were then washed twice with PBS and incubated for 24 h in a 30% sucrose solution. Tissues were then rapidly frozen in OCT embedding media in an 100% ethanol and dry-ice slurry and stored at −80 °C. Tissue sections of 20 μm were prepared using a Leica CM1950 cryostat. Tissue sections were incubated in blocking buffer for 1 h and stained overnight at room temperature with fluorescently conjugated antibodies and mounted with Fluoromount-G^™^ Mounting Medium. Imaging was performed using a Leica Stellaris8 confocal microscope with 20x 0.75NA (Fig. 4) or 40x 1.3NA (Figs. 5–6) oil objectives.

The following antibodies or reagents were used: CD45.1–BV421 (clone A20; BioLegend, Cat#110732), CD45.2–R718 (clone 104; BD Biosciences, Cat#567585), CD4–BV480 (clone RM4–5; BD Biosciences, Cat#565634), pS6–conjugated in house (clone D57.2.2E; Cell Signaling Technology, Cat#99457), CD103–conjugated in house (polyclonal; R&D Systems, Cat#AF1990), CD68–mFluor Violet 610 (clone FA-11; Novus Biologicals, Cat#NBP2–33337MFV610), CD4–conjugated in house (clone RM4–5; BioLegend, Cat#100506), KLRG1–conjugated in house (clone 2F1; Invitrogen, Cat#16-5893-82), CD68–BV421 (clone FA-11; BioLegend, Cat#137042). E-cadherin-conjugated in house (clone DECMA-1, Cat#147301). Antibody labeling kits: Mix-n-Stain^™^ CF514 (Biotium, Cat#92331), CF555 (Biotium, Cat#92274), CF594 (Biotium, Cat#92448), CF633 (Biotium, Cat#92277), CF660C (Biotium, Cat#92280).

### Histo-cytometry

Histo-cytometry analysis was performed as previously described with minor adaptations^12,39,67^. Fluorescence spillover in confocal images was corrected using the Leica Channel Dye Separation module. Single-color controls were prepared by mixing fluorescently conjugated antibodies with Fluoromount-G^™^ Mounting Medium on slides, followed by imaging. Cell segmentation was conducted using the Imaris surface creation module based on NucSpot nuclear staining. Mtb and E-cadherin were detected using the Imaris spot creation function applied to PPD or E-cadherin staining, respectively. For mixed bone marrow chimeras (BMCs), the Channel Arithmetics Imaris XTension was used to resolve overlapping CD45.1 and CD45.2 signals (Voxel gating). This approach addressed the issue of signal bleed caused by the close proximity of adjacent cells, ensuring accurate distinction between the two markers. Here, two distinct channels, CD45.1_clear and CD45.2_clear, were generated through signal subtraction. CD45.1_clear was defined as “CD45.1 − CD45.2”, and CD45.2_clear was defined as “CD45.2 − CD45.1”. (Suppl Fig. 6A). Additionally, voxel gating was performed to identify CD103 + CD45.1 + CD4 T cells, as previously described.^91^ Briefly, a composite channel (CD103_comp) was generated by combining voxels surpassing defined thresholds for CD103, CD45.1, and CD4 (Suppl Fig. 6B). Object statistics were exported to Excel (Microsoft) and imported to FlowJo Software for hierarchical gating and phenotypical characterization (example shown in Suppl Fig. 3A–B). For the BMCs, gating was performed on the new channels (CD45.1_clear, CD45.2_clear and CD103_comp) to accurately quantify CD45.1, CD45.2 and CD103 CD4 T cells respectively. CSV files containing annotated cell phenotypes were exported from FlowJo and imported to CytoMap for spatial analysis.^68^ There, a virtual raster scan was performed to subdivide the tissue into neighborhoods containing information about the cellular composition and density. The neighborhoods were then clustered into regions with similar cellular composition (e.g. densities of CD4^+^ T cells, macrophages, PPD + macrophages) enabling the identification of discrete areas within the tissue (e.g. lesion areas, unaffected lung areas). For the analysis in Fig. 6E, multiple images containing both lesion tissue and adjacent unaffected lung were collected from each mouse and pooled for analysis. Because images differed in size and in the proportion of lesion versus unaffected tissue, cell densities were normalized to the area of each region. For the analysis of Mtb microenvironments, CytoMAP was used to define PPD-centered neighborhoods including areas within 100 μm from a PPD spot. For visual clarity, presented images were manipulated using Imaris and PowerPoint (Microsoft), with identical adjustments applied across all experimental groups.

### Bioinformatic analysis of public datasets

#### Nonhuman primate granuloma dataset

**Publicly available single-cell RNA sequencing data** from *Mycobacterium tuberculosis* (Mtb)-infected cynomolgus macaque granulomas (**GEO accession: GSE200151**) were reanalyzed. Raw gene count matrices and accompanying cell annotation metadata were obtained from the Gene Expression Omnibus. Data were imported into a Single-CellExperiment object in R, and log-normalized using the *scuttle* package to correct for sequencing depth variation.

#### Human blood and lung CD4^+^ αβ T cells

**Human single-cell transcriptomic data were accessed through the CELLxGENE Census (census version “2024-07-01″) via the official Python API. Cells were filtered to include only primary CD4**^+^ **αβ T cells (**cell_type == 'CD4-positive, alpha–beta T cell' and is_primary_data == True) derived from blood and lung tissues. Cells generated using the 10x Genomics 5′ assay or Smart-seq2 were excluded. The final dataset comprised 403,937 blood-derived and 187,852 lung-derived CD4^+^ αβ T cells.

Gene selection was performed using the pp.highly_variable_genes function in *Scanpy* to identify the top 1,200 most variable genes. Data integration and downstream analysis were carried out using *scvi-tools*. A variational autoencoder model (scvi.model.SCVI) was trained with categorical covariates for donor and suspension type, and continuous covariates for mitochondrial and ribosomal gene percentages. Differential gene expression was computed using the model.differential_expression method. Genes with a false discovery rate (FDR) < 0.05 were considered statistically significant.

Cells were classified as ITGAE^+^, KLRG1^+^, or CD4^+^ if they expressed ≥ 1 raw count of *ITGAE*, *KLRG1*, or *CD4*, respectively.

All analyses were conducted using Python and R on an NVIDIA H100 GPU with 80 GB HBM3 memory, pytorch precison of float32 matrix multiplications was set to high. The full analysis pipeline and package dependencies are available at: https://github.com/INFIMM-Bioinformatics/TB-cd103-cd4-Tcells.

### Statistical analyses

All graphical visualizations and statistical tests were done using GraphPad Prism v10. Statistical differences between two treatments or groups were inferred using Students *t*-test, either paired or unpaired, depending on the experimental layout. Two-way Analysis of Variance (ANOVA) with Tukey’s Multiple Comparison test or One-Way Analysis of Variance (ANOVA) using Dunnett’s multiple comparison test (comparing to control mice only) or Tukey’s Multiple Comparison test (comparing across all groups) were used to evaluate significant differences between more than two groups. CFU values were log-transformed before statistical analysis. The specific statistical test, including corrections used, is stated in the figure legends. A p-value above 0.05 was considered not significantly different.

## Supplementary Material

1

## Data Availability

The data that support the findings of this study are available from the corresponding author upon request. There are no restrictions on data availability.

## Appendix A. Supplementary data

Supplementary data to this article can be found online at https://doi.org/10.1016/j.mucimm.2026.02.001.
