# Supplementary material for: Expression of CD103 facilitates localization and activation of CD4+ T cells within Mycobacterium tuberculosis lung-lesions
Source: Mucosal Immunol. Author manuscript; Available in PMC 2026 Jun 22. (PMC13284540; doi:10.1016/j.mucimm.2026.02.001)
Supplement: 1 [file NIHMS2179680-supplement-1.pdf]

Supplementary Fig 1

A)

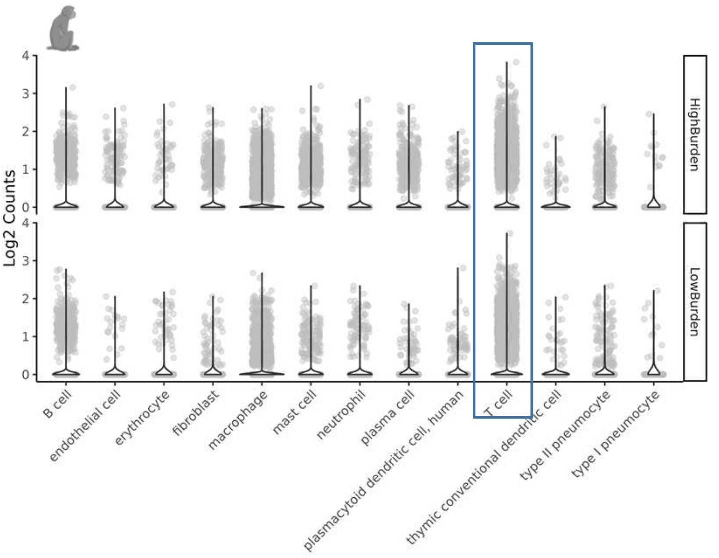

B)

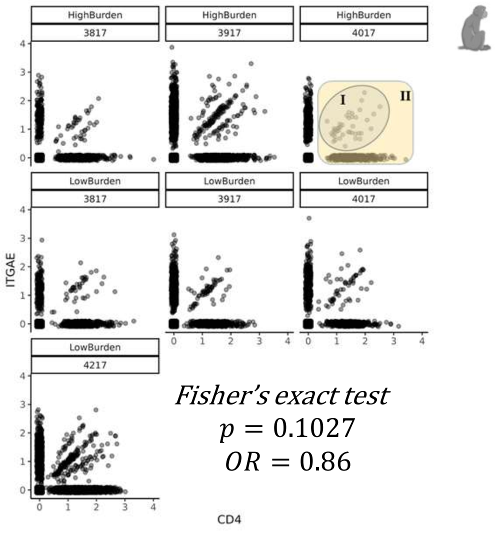

C)

(Lung granuloma after MtB infection)

	Phenotype	NHP		Human	
		Wk4 (%)	Wk10 (%)	Lung (%)	Blood (%)
0	KLRG1+ ITGAE+	0.2	0.4	0.7	0.5
1	KLRG1+ ITGAE-	2.2	4.0	5.1	4.2
2	KLRG1- ITGAE+	12.4	10.4	11.7	7.4
3	KLRG1- ITGAE-	85.3	85.2	82.4	88.0

Supplementary Figure 1

(A) ITGAE expression across cell types in monkey granulomas at week 10. Violin plots showing the log2-normalized expression levels of ITGAE across different cell types in granulomas from week 10 samples. Each point represents an individual cell, with jittered points overlaid to show the distribution of expression values. The data is stratified by granuloma burden (High Burden and Low Burden). (B) Correlation between CD4 and ITGAE expression in T cells from granulomas at Week 10. Scatter plots showing the log2-normalized expression levels of CD4 and ITGAE in T cells, stratified by granuloma burden (High Burden and Low Burden) and donor ID. Each point represents an individual cell. We used Fisher's exact test to assess whether the frequency of ITGAE$^+$ CD4$^+$ T cells differed between granulomas with high versus low burden. Among 2,023 CD4$^+$ T cells from high-burden granulomas, 237 (11.7%) were ITGAE$^+$, compared to 343 of 3,336 CD4$^+$ T cells (10.3%) from low-burden granulomas. This difference was not statistically significant (Fisher's exact test, P = 0.1027; odds ratio = 0.86, 95% CI: 0.72–1.03). (C) KLRG1 and ITGAE expression in CD4$^+$ T Cells from lung granulomas in NHPs and Humans. The table summarizes the distribution of CD4$^+$ T cells across four phenotypic subsets defined by KLRG1 and ITGAE expression. Data are shown for non-human primates (NHPs) at Week 4 and Week 10 following M.tb infection, and for human lung and blood samples. Phenotypic classification was based on the presence or absence of KLRG1 and ITGAE expression, with positivity defined as detection of at least one count.

Supplementary Fig 2

A)

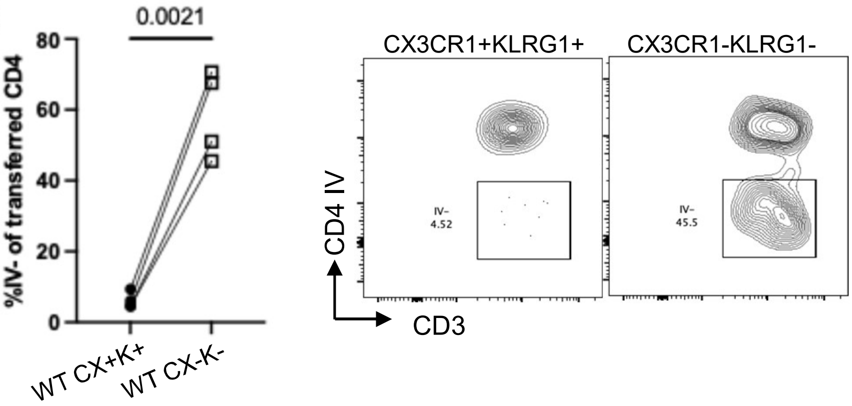

Supplementary Figure 2

(A) Trafficking (percent iv-negative) of CX3CR1+KLRG1+ vs CX3CR1-KLRG1- lung CD4^{+} T cells isolated from wt C57BL/6 mice at Day 28 post-Mtb infection and adoptively transferred into congenically marked, infection-matched recipients.

Supplementary Figure 3

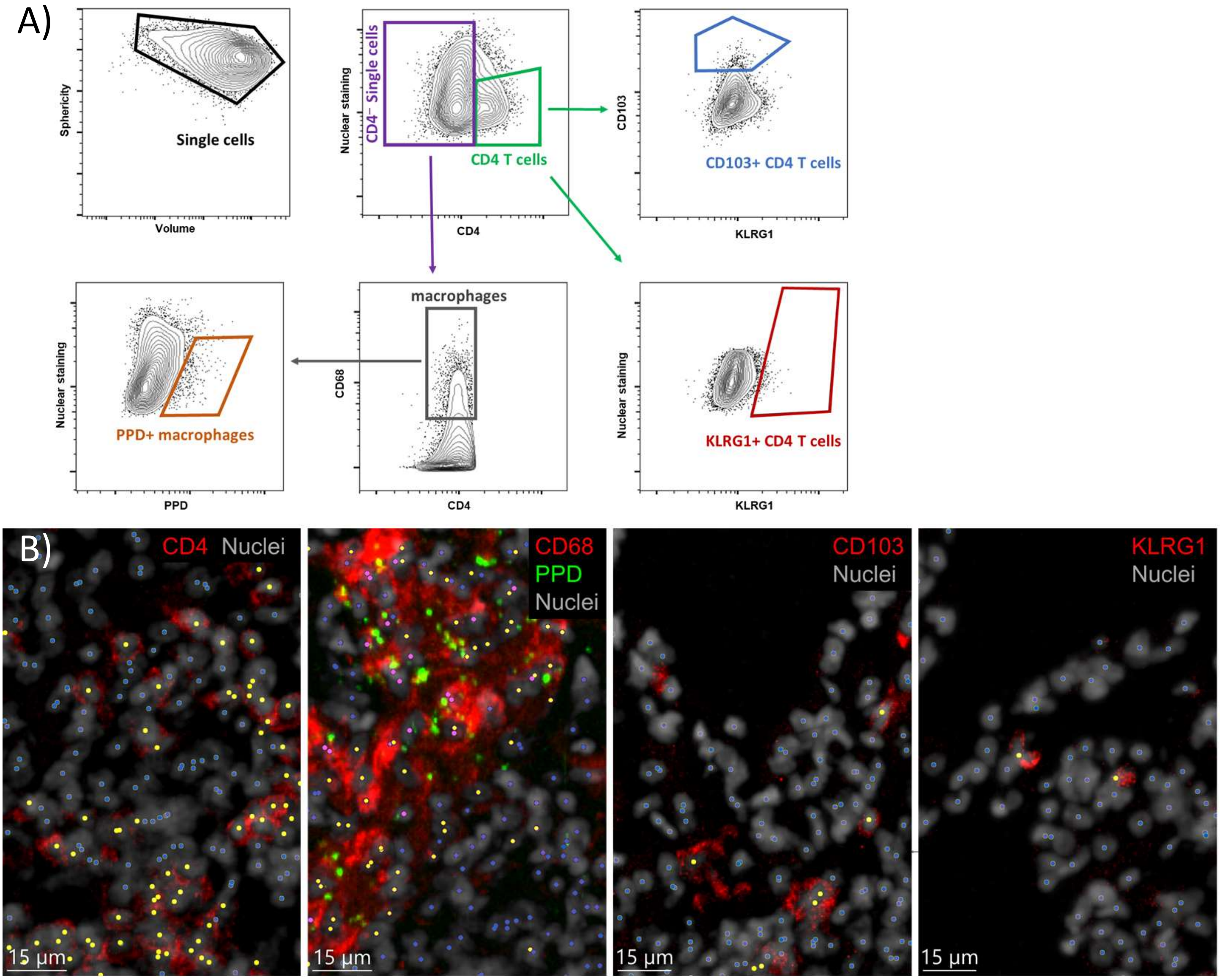

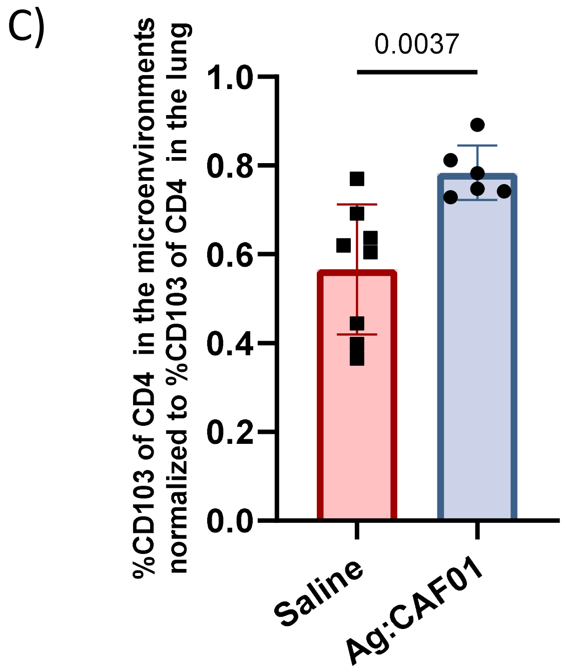

Supplementary Figure 3. Histocytometry gating strategy of cells within infected lungs & normalization of CD103+ CD4 T cells within Mtb microenvironments among saline and Ag:CAF01-immunized mice.
(A) Example of histocytometry gating strategy and phenotypical characterization of cells within infected lungs. (B) Visualization of histocytometry gating overlaid on confocal images. Cell nuclei are shown in grey, and the relevant markers are shown in red or green (for PPD). Blue dots represent negative cells, yellow dots indicate positive cells, and magenta dots denote double-positive $CD68^{+}$ PPD^{+} cells. (C) Normalized frequencies of CD103+ CD4 T cells within Mtb microenvironments in saline and Ag:CAF01-immunized mice. Frequencies of CD103+ CD4 T cells in Mtb microenvironments (as defined in Figure 4D) were normalized to their corresponding frequencies in the total lung for each section. Statistical analysis was performed using a t-Test with Welch’s correction

Supplementary Figure 4

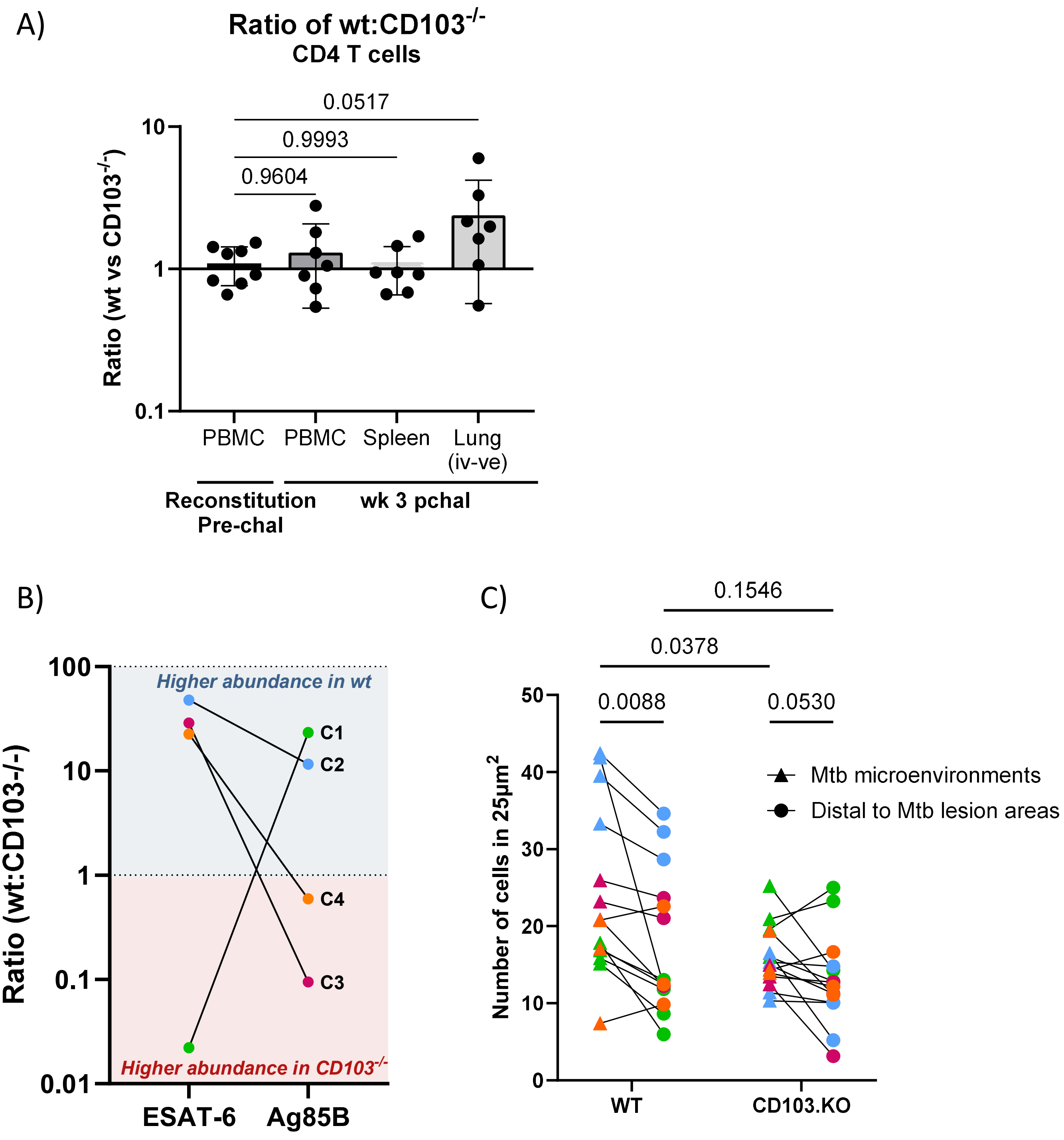

Supplementary Figure 4

(A) Mixed BMC mice were bone marrow reconstituted with a 1:1 ratio of WT:CD103-/- cells. Ratios of WT:CD103-/- CD4$^+$ T cells were then calculated in PBMCs pre-Mtb challenge (after immunizations and just prior to Mtb challenge) as well as in PBMCs, spleen and lung parenchyma (iv-ve) at week 3 post-challenge. P-values were calculated using a one-way ANOVA followed by Dunnett's multiple comparisons test. (B) The ratio of wt to CD103$^{-/-}$ (wt:CD103$^{-/-}$) ESAT-6- and Ag85B-tetramer specific cells within each chimeric mouse used for histocytometry were calculated. Hence, a ratio above 1 reflects a higher abundance among wt chimeric donor cells, whereas a ratio below 1 signifies a higher frequency of CD103$^{-/-}$ Tet+ cells. Individual mice are tagged and color-coded. Mice C1, C3 & C4 show bidirectional skewing of Mtb-specific cells, such a single mice harbour Ag85B- or ESAT-6-specific responses skewed in opposite directions of wt vs CD103$^{-/-}$ chimeric CD4 T cell dominance. Only mouse C2 exhibit unidirectional skewing of both tetramer specificities towards wt cells. (C) Figure 5H showing densities of WT or CD103-/- CD4+ T cells in Mtb microenvironments or distal to Mtb areas within lesions. p-values are calculated using Repeated Measures two-way ANOVA with Geisser-Greenhouse correction. Lines connect values within same lesions. Color-coded as in Suppl Figure 5B for clarity.

Supplementary Figure 5

A)

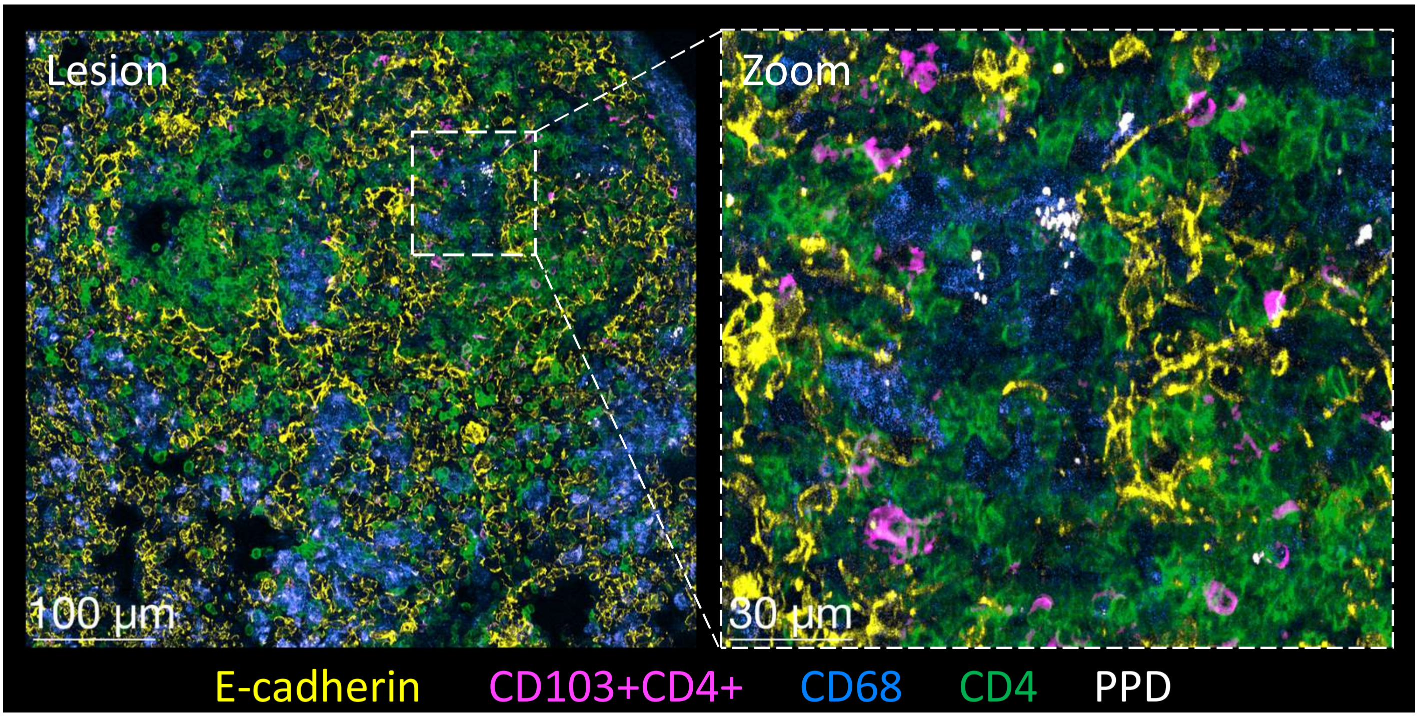

B)

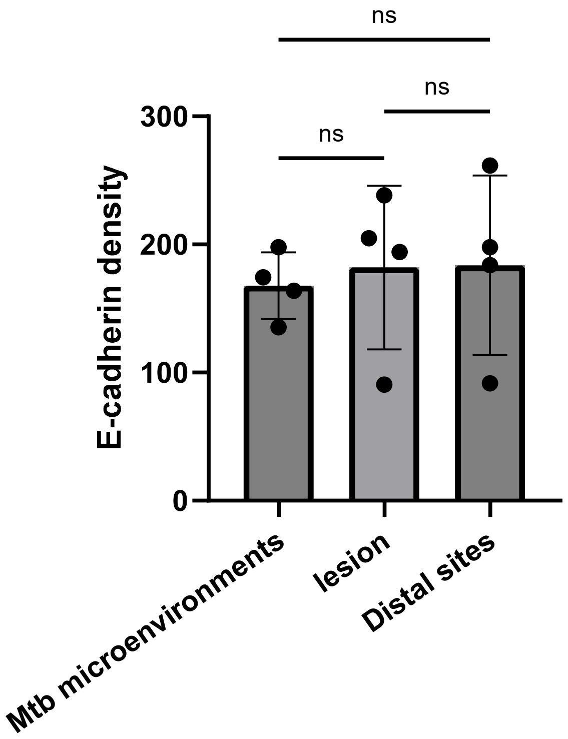

Supplementary Figure 5. E-cadherin is available for CD103 ligation in Mtb infected lung tissue and uniformly present across lung regions during Mtb infection in Ag:CAF01 immunized mice.
Mice were immunized with two doses of Ag:CAF01, administered four weeks apart, and challenged with Mtb five weeks after the final dose. Lungs were harvested at 21 days p.i. for analysis. (A) Representative confocal image showing staining for E-cadherin, $CD4^{+}$ T cells, $CD103^{+}CD4^{+}$ T cells, CD68 and PPD (left) with zoomed in images (right). (B) Histo-cytometry quantification of E-cadherin density in different lung regions, calculated as spots per 50µm radius neighborhood. Each data point represents one mouse (n = 4). Statistical analysis was performed using one-way ANOVA (all $p > 0.999$).

Supplementary Figure 6

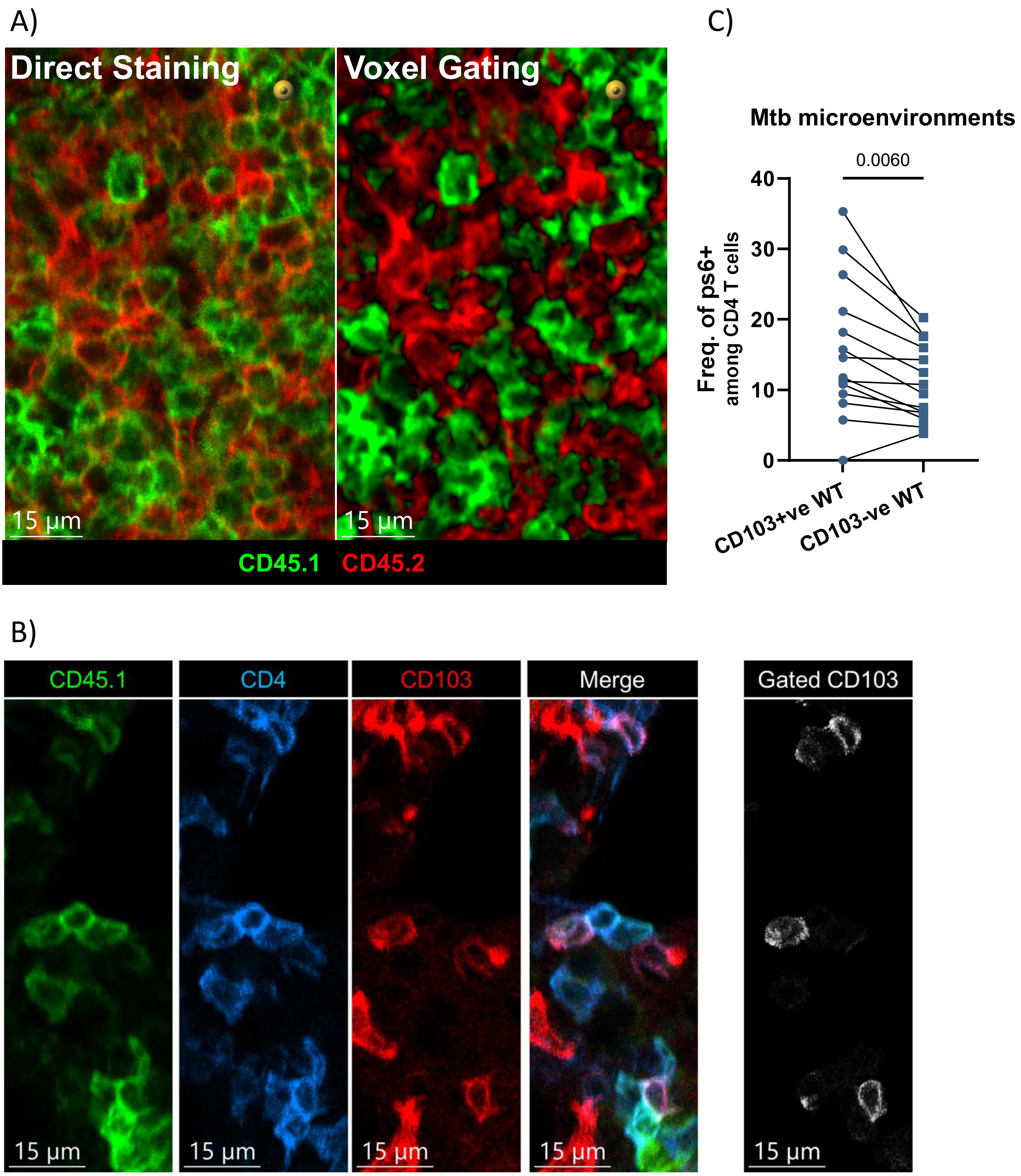

Supplementary Figure 6

(A) Representative images showing CD45.1 and CD45.2 signals before (left) and after voxel gating (right). Voxel gating was performed as described in the Methods to resolve overlapping signals between adjacent cells. (B) Voxel gating strategy used to identify $CD103^{+}$ $CD45.1^{+}$ $CD4^{+}$ T cells, as described in the Methods. CD45.1, CD4, and CD103 channels are shown individually and merged, followed by the composite CD103 channel generated by voxel gating. (C) Mtb microenvironments were identified at CytoMap by creating 100µm-radius Mtb-centered neighborhoods. Graph shows frequencies of pS6+ out of CD103+ or CD103- WT $CD4^{+}$ T cells. P-value was calculated using paired t-test.
